# Supplementary material for: Single-molecule RNA sizing enables quantitative analysis of alternative transcription termination
Source: Nat Commun. 2024 Feb 24;15:1699. doi: 10.1038/s41467-024-45968-8 (PMC10894232; doi:10.1038/s41467-024-45968-8)
Supplement: Supplementary file 1 — Supplementary Information [file 41467_2024_45968_MOESM1_ESM.pdf]

Supplementary Material for

Single-Molecule RNA Sizing Enables Quantitative Analysis of Alternative Transcription Termination

Patiño-Guillén, G., Pešović, J., Panić, M., Savić-Pavićević, D., Bošković, F.*, Keyser, U.F.*

Correspondence to: fnb24@cam.ac.uk , ufk20@cam.ac.uk

This PDF file includes:

→ Supplementary Figures 1 to 29
→ Supplementary Tables 1 to 8

Supplementary Figure 1.

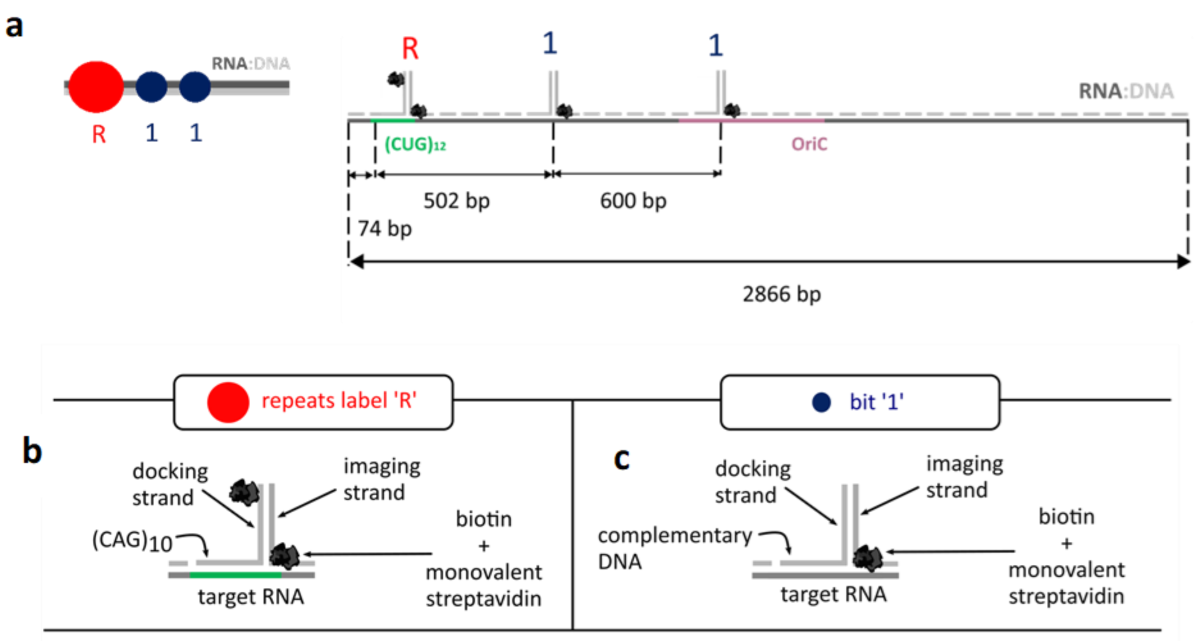

Supplementary Figure 1. Design of RNA ID. **a** The position of the repeats label 'R' and the '1' bits within the RNA ID is shown. **b** 'R' represents a DNA CAG_{10} oligonucleotide (docking strand) that binds to the CUG repeats, this oligo has an overhang sequence with 3' biotin that binds to a complementary strand (imaging strand) with another 3' biotin, enabling the overall binding of two monovalent streptavidins to RNA ID. **c** The RNA ID is decorated with '1'. Two '1' bits are included in the RNA ID design to produce a distinguishable signal. '1' bits lack biotin on the docking strand, which enables the binding of only one monovalent streptavidin per bit, providing a discriminatory signal in nanopore recordings.

Supplementary Figure 2.

a

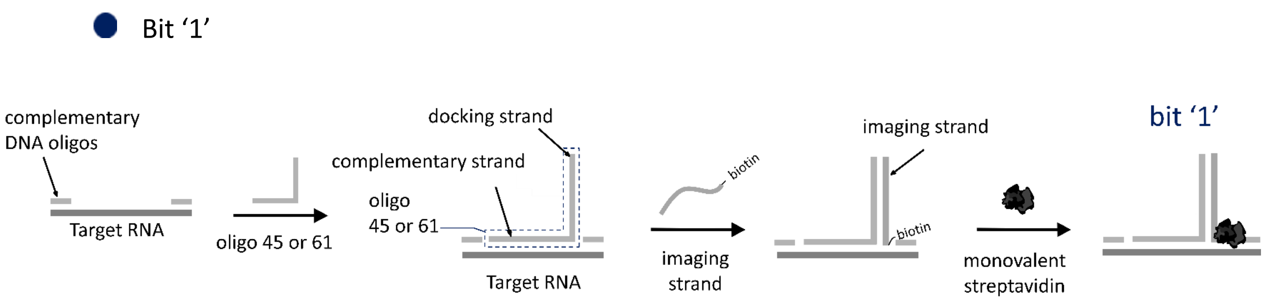

b

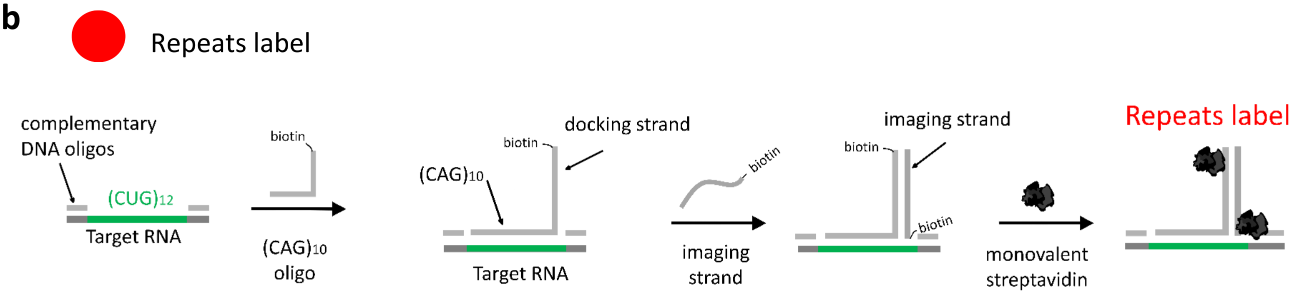

c

Oligo name	Sequence (5'→ 3')
Docking strand	TTTGGATATCACTCATTAGTGGT
Imaging strand	ACCACTAATGAGTGATATCC/3'-biotin/

Supplementary Figure 2. Assembly of RNA ID. **a** Detailed assembly of '1' bits. The sequence of each '1' bit can be found in Supplementary Table 2, corresponding to oligos 45 and 61. **b** Detailed assembly of repeats label 'R', sequence can be found in Supplementary Table 2 (oligo 76). **c** Table shows the sequence of docking strand overhang and its complementary oligo (imaging strand) which enable streptavidin binding.

Supplementary Figure 3.

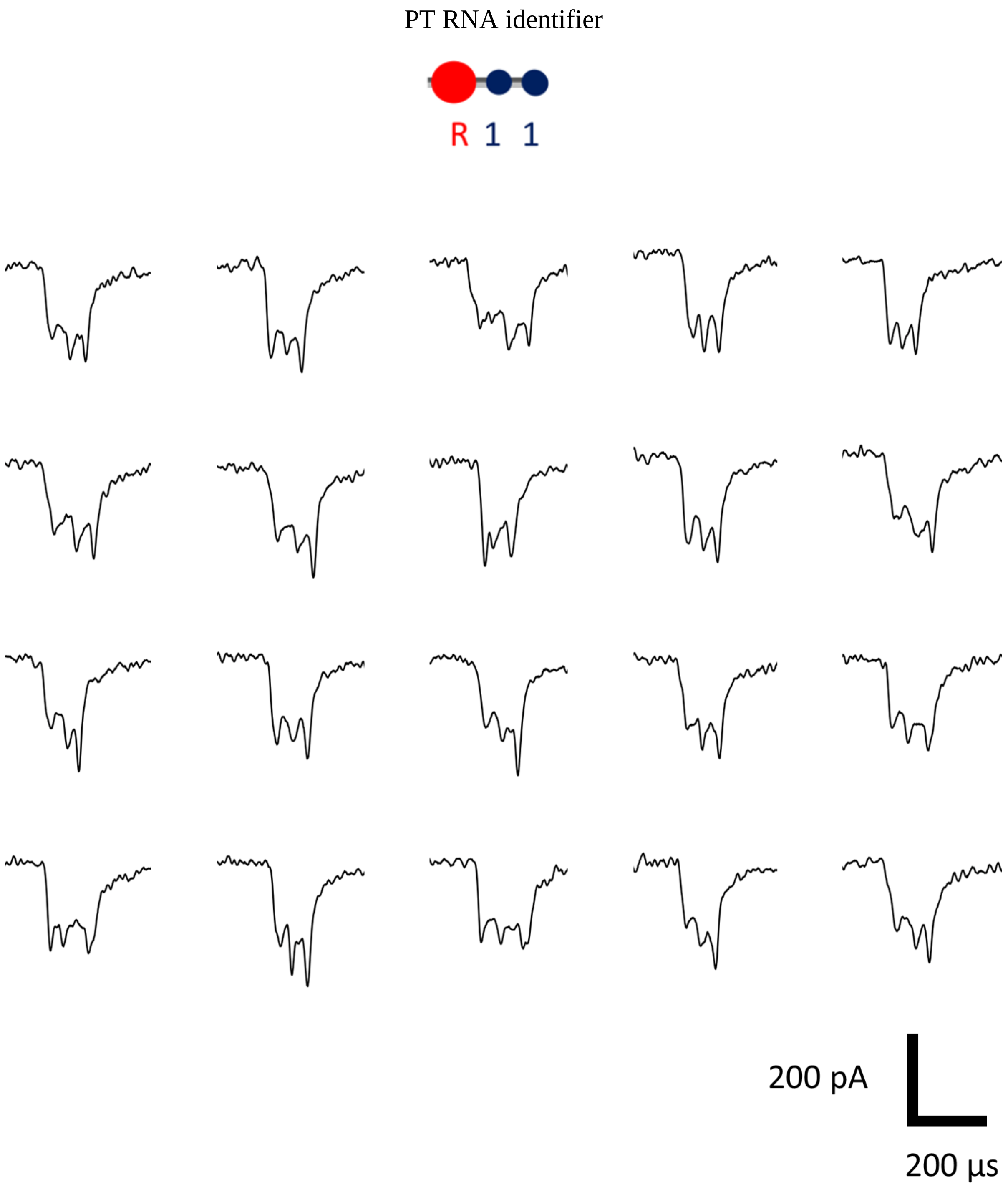

Supplementary Figure 3. Nanopore translocation events of RNA identifier from premature transcription termination (PT). These are the first 20 unfolded translocation events detected of PT RNA IDs. Nanopore event variability can be accounted to the physical configuration-dependent RNA ID transport[1].

Supplementary Figure 4.

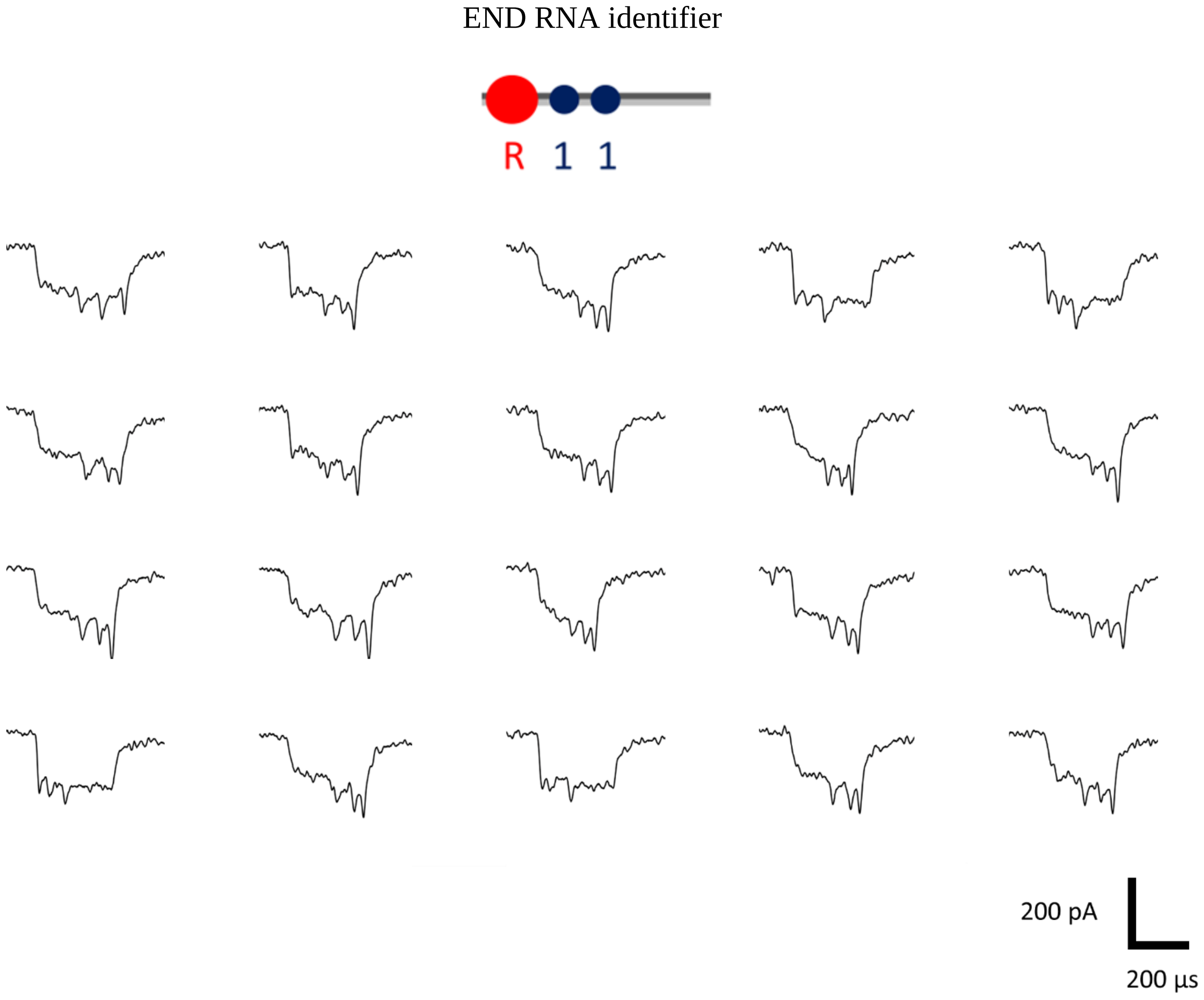

Supplementary Figure 4. Nanopore translocation event of RNA identifier from transcription of the complete linear DNA (END). T7RNAP falls off at the end of the linear template causing transcription termination. These are the first 20 unfolded translocation events detected of END RNA IDs.

Supplementary Figure 5.

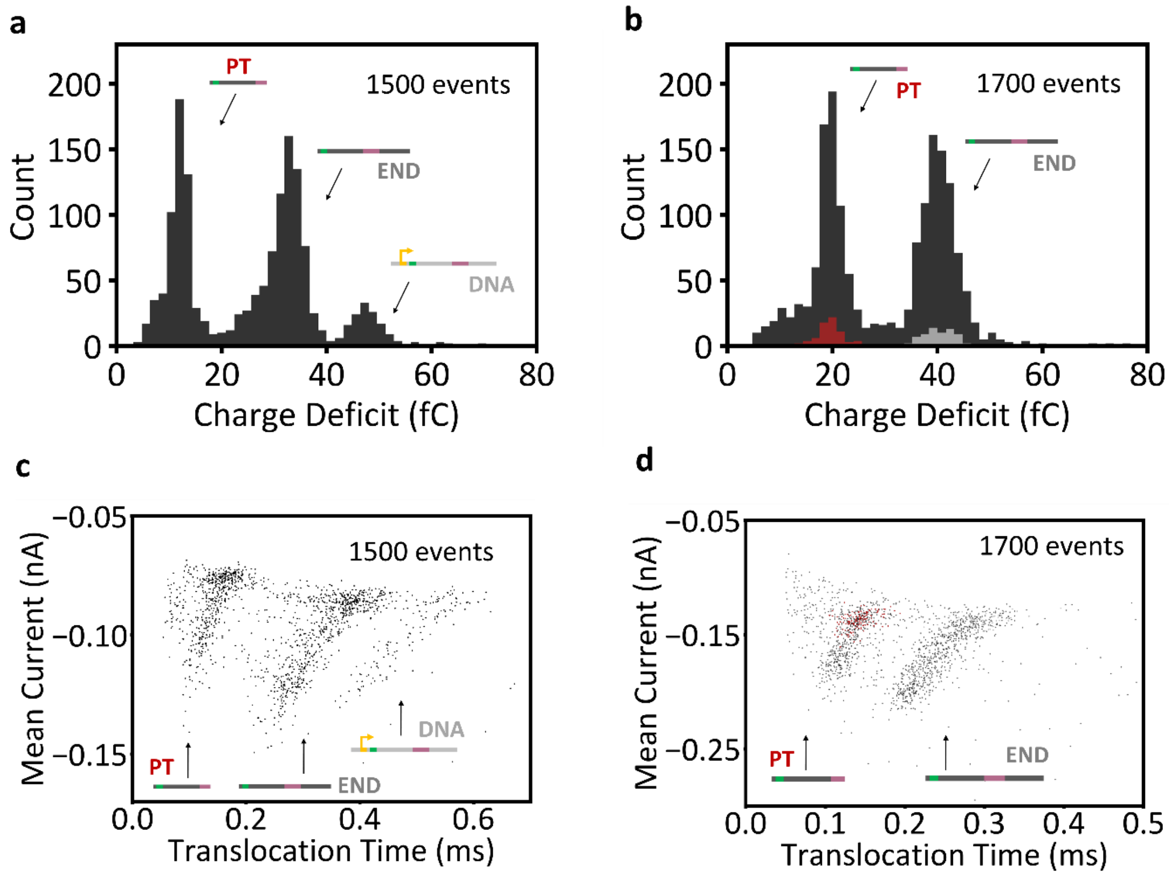

Supplementary Figure 5. Characterization of RNA ID using charge deficit, mean current and translocation time. **a** Histogram of the charge deficit of RNA ID still in the presence of the linear DNA template. Histogram shows 3 distributions, the one with the lowest charge deficit is ascribed to premature termination (PT), the middle distribution corresponds to transcription of the full linear DNA (END) and the distribution furthest to the right is ascribed to the linear DNA template. This distribution (composed of 1500 events) includes translocations of molecules with multiple conformations, which include folded events, constructs with knots and unfolded events. **b** After treatment with DNase I, it can be seen how the distribution furthest to the right, ascribed to DNA, is removed. Unfolded translocations of both PT (red) and END (gray) RNA IDs (presented in Figure 2c) describe the entire sample, despite their conformation, while enabling single-molecule sizing. These distributions correspond to the same nanopore measurement presented in Figure 2. **c** Scatter plot of mean current against translocation time shows three distinct distributions, attributed to PT RNA IDs, END RNA IDs, and the linear template (from left to right). Unfolded molecules take longer to translocate through the pore than folded molecules but cover less cross-sectional area of the pore while translocating,

producing a less significant drop in ionic current for longer times. Plotting events with different conformations produces this type of distribution, with unfolded events at the top, and folded events at the bottom of each distribution. **d** Selection of unfolded events (in the sample treated with DNase I) is performed to describe each distribution. Unfolded events were found at the top of each distribution, and they are representative of the whole sample.

Supplementary Figure 6.

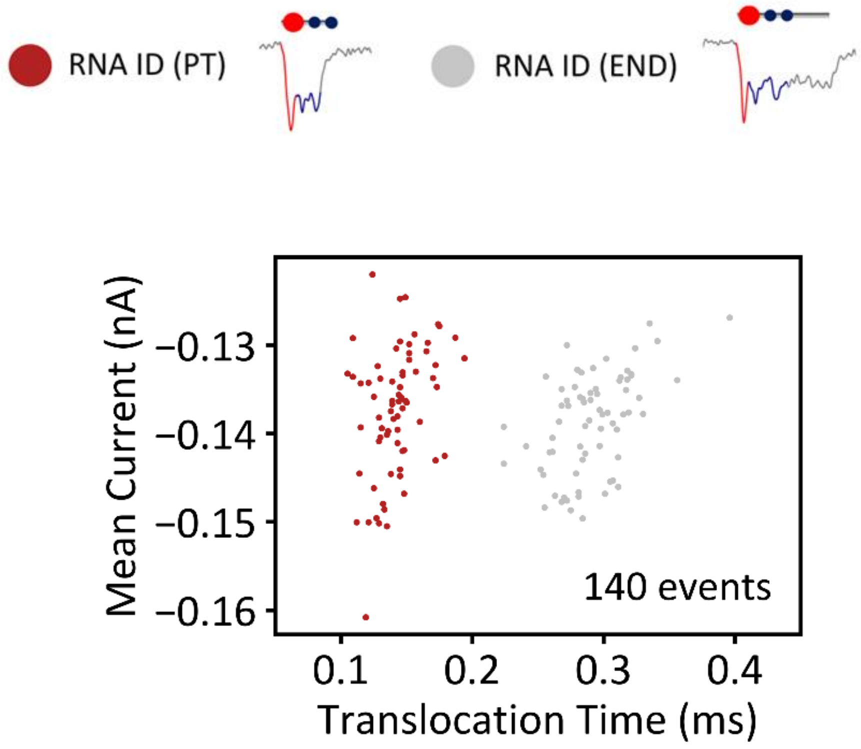

Supplementary Figure 6. Scatter plot of mean current against translocation time for unfolded END (red) and PT (gray) RNA IDs, which depicts two distinct populations. This demonstrates that plotting these two parameters for transcripts with different termination sites enables their distinction.

Supplementary Figure 7.

a

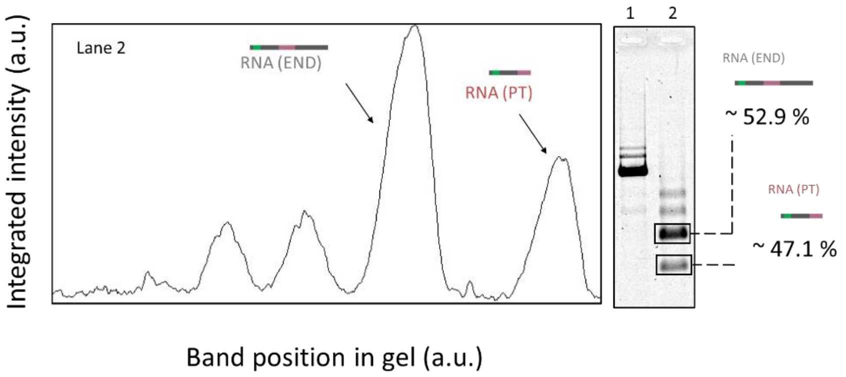

b

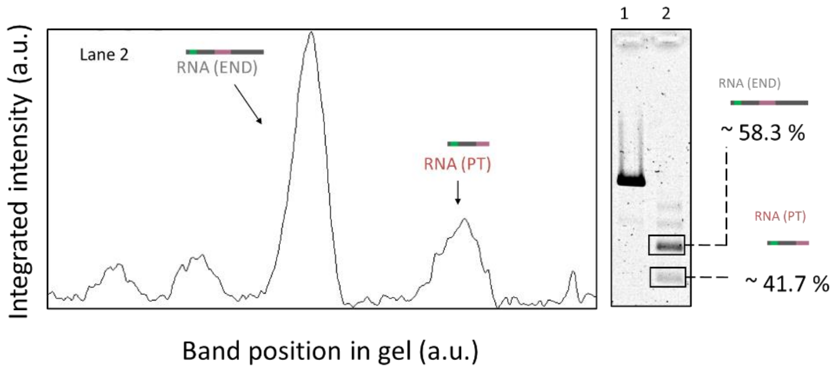

For **a**

Lane	Molecule	Intensity profile (a.u.)	Normalized intensity profile (a.u./bp)
1	RNA (PT)	8181.711	5.389
	RNA (END)	17336.317	6.049

For **b**

Lane	Molecule	Intensity profile (a.u.)	Normalized intensity profile (a.u./bp)
1	RNA (PT)	5107.296	3.364
	RNA (END)	13481.004	4.703

Supplementary Figure 7. Quantitative analysis of premature transcription termination in OriC using agarose gel electrophoresis. **a** The gel shows the linearized construct in lane 1 and the DNase I treated transcripts in lane 2. The two topmost bands in lane 2 are attributed to RNA side products. The two lower bands correspond to END RNA and PT RNA, from top to bottom.

The intensity profile of these two bands was plotted and the area of each peak was computed. The peak areas were normalized by the number of base pairs of each transcript to obtain an estimate of transcript abundance. Gel suggests premature transcription termination of ~ 47.1 % in OriC. The same experimental procedure and analysis described was repeated to evaluate variability in RNA abundance quantification. Gel suggests premature transcription termination of ~ 41.7 % in OriC, indicating good reproducibility. Lane 1 – linear DNA, Lane 2 - DNase treated transcripts. Gel: 1 % (w/v) agarose, 1 × TBE, 0.02% sodium hypochlorite.

Supplementary Figure 8.

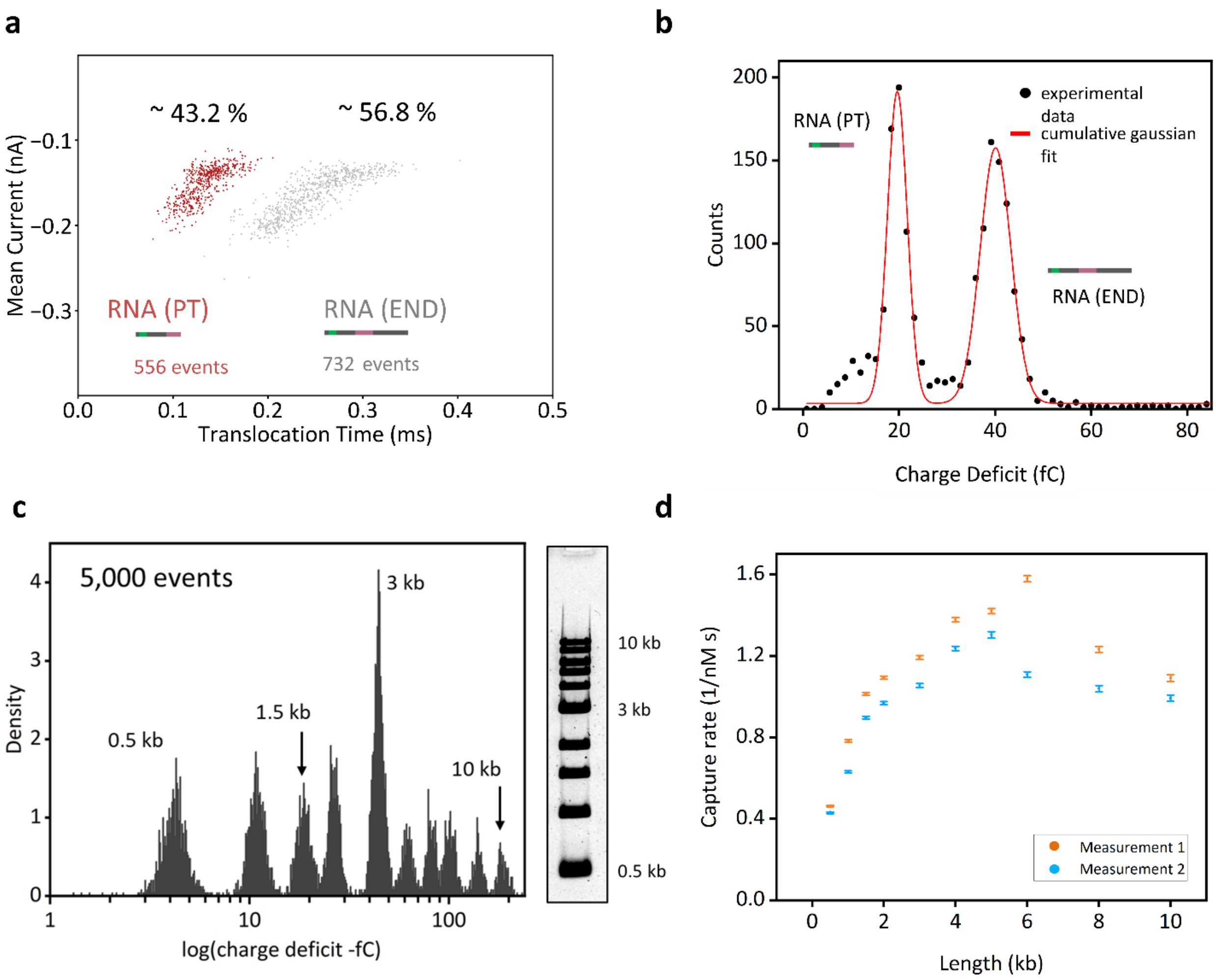

Supplementary Figure 8. Quantitative study of PT RNA IDs and END RNA IDs events detected in nanopore sensors. **a** As illustrated in Supplementary Figure 5c and Supplementary Figure 5d, scatter plots of mean current against translocation time can be used to identify RNA IDs from transcripts with different termination sites. Scatter plot exhibits two distributions: the one from the left is ascribed to PT RNA ID and the distribution at the right corresponds to END RNA ID. The events within each of these distributions were counted by establishing lower and upper limits in charge deficit, the rest of the events were discarded. The upper limit for RT RNA IDs was 25 fC, and the lower limit was 15 fC. For END RNA IDs the upper limit was 50 fC and the lower limit was 30 fC. 556 events were detected for RT RNA IDs and 732 events were identified for END RNA IDs, suggesting ~ 43.2% termination in OriC. **b** Cumulative Gaussian fit of PT and END RNA IDs. The lowest charge deficit peak is ascribed to premature termination (PT) and the middle peak corresponds to full-length RNA transcript (END). Comparison of the areas of PT RNA ID and END RNA ID fits suggest ~44% transcription

termination. **c** A 1kb DNA ladder (NEB) containing DNA molecules from 0.5 kb to 10 kb was used to study capture rate variability in our nanopore system ascribed to a difference in length-dependent capture rate[2,3]. The concentration is known for the DNA of each length, based on information provided by the manufacturer. Characterization of the DNA ladder in nanopores was performed in duplicate. **d** Capture rate for each DNA molecule length included in the DNA ladder for both measurements.

Supplementary Figure 9.

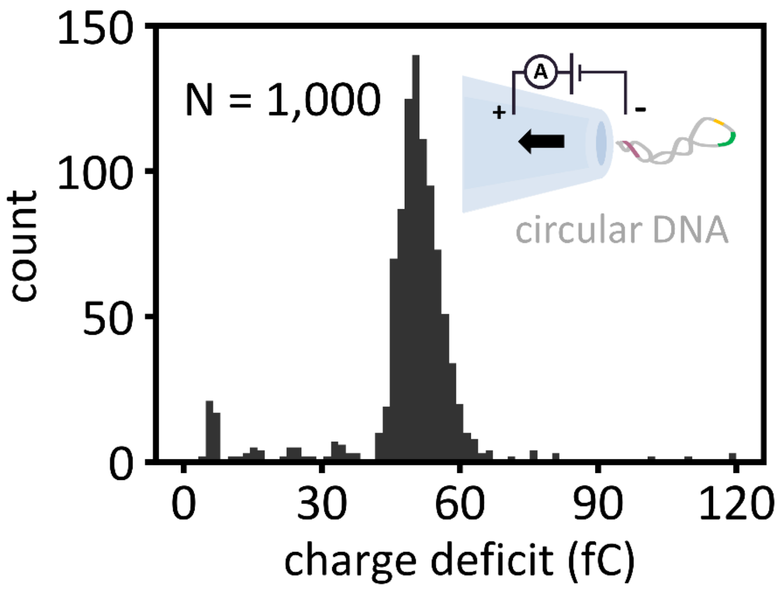

Supplementary Figure 9. The 3.1 kbp circular DNA used as the template for rolling circle transcription was characterized using nanopore sensing. The charge deficit of the translocation events shows a unimodal distribution, demonstrating the presence of a single DNA construct. This also confirms RNA is produced from circle rolling transcription of the 3.1 kbp circular DNA, and not by transcription of DNA dimers or trimers of the 3.1 kbp DNA which could be a possible interpretation of agarose gel electrophoresis. Here we also showcase the clarity nanopore sensing provides for the elucidation of DNA identity.

Supplementary Figure 10.

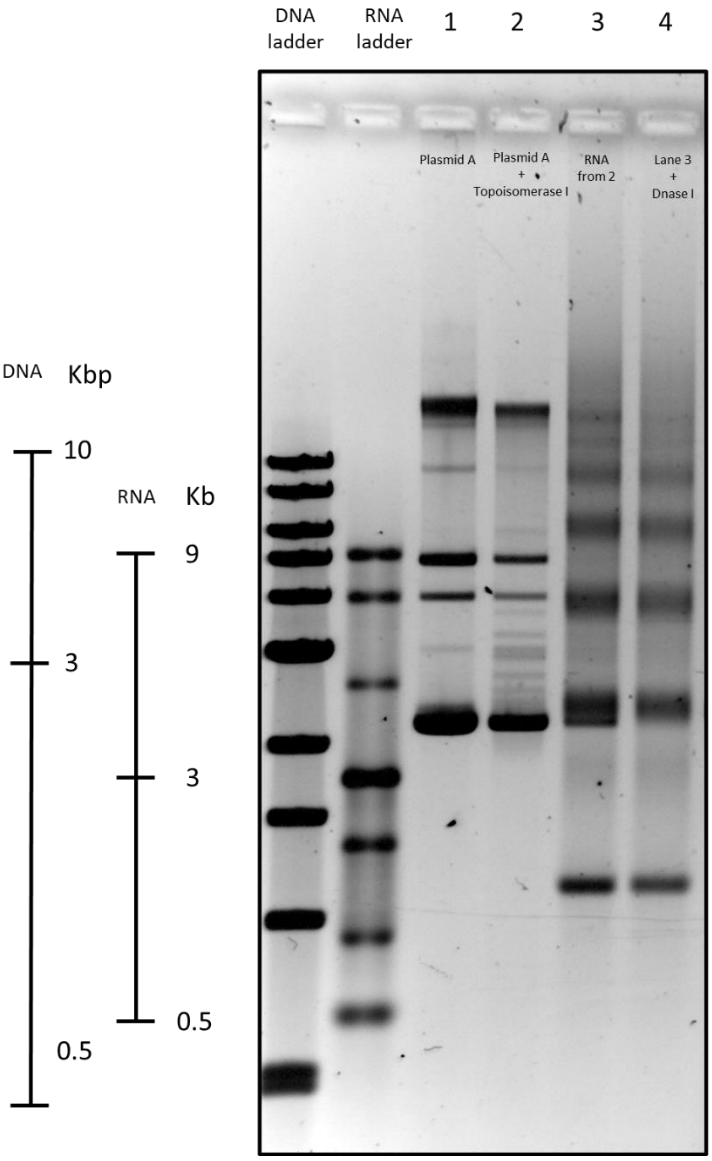

Supplementary Figure 10. Rolling circle transcription of circular DNA construct. DNA ladder and ssRNA ladder are included on both sides of the gel. Lane 1 – Circular plasmid with 12 CTG repeats (sequence of circular plasmid in Supplementary Table 1). The multiple bands are ascribed to the physical configurations that supercoiled DNA has while being electrophoretically driven through the agarose gel. Lane 2 – circular plasmid from lane 1 treated with Escherichia coli Topoisomerase I to induce plasmid relaxation. Lane 3 – RNA from transcription of Topoisomerase I treated circular plasmid in lane 2. Lane 4 – RNA from lane 3 treated with DNase I. From DNase I treatment, DNA band located at ~3 kbp (DNA) is removed. The rest of the bands, ascribed to RNA products of the multiple transcription cycles, remain.

Supplementary Figure 11.

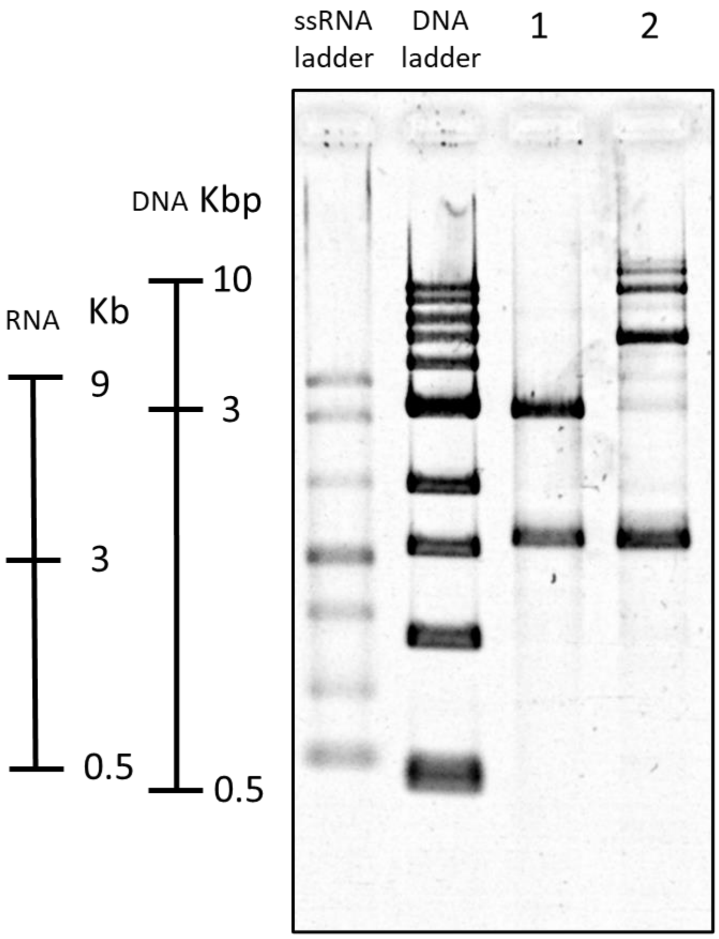

Supplementary Figure 11. RNA ID of transcripts from transcription of linear DNA construct and rolling circle transcription of circular DNA construct (sequence in Supplementary Table 1). The agarose gel shows a DNA ladder and ssRNA and DNA ladder on the left side. Lane 1 – RNA ID assembled from RNA from transcription of linear DNA template. Lane 2 - RNA ID assembled from transcripts of rolling circle transcription. Gel: 1 % (w/v) agarose, 1 × TBE, 0.02% sodium hypochlorite.

Supplementary Figure 12.

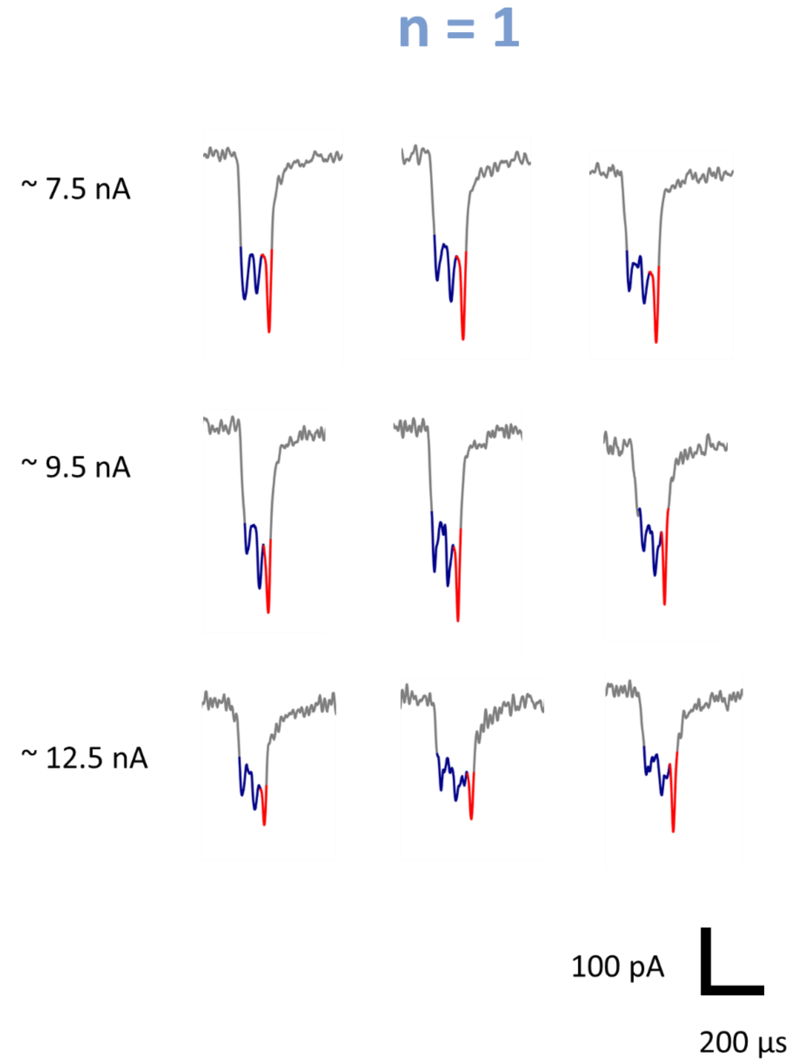

Supplementary Figure 12. N = 1 example events. Example nanopore events of RNA IDs produced from one transcription cycle (N = 1) measured in pores with different sizes, which exhibit different signal to noise ratio. Variation in pore size can be inferred from changes in the ionic current baseline. All measurements were performed under the same applied voltage of 600 mV. Larger pores have a larger ionic current baseline.

Supplementary Figure 13.

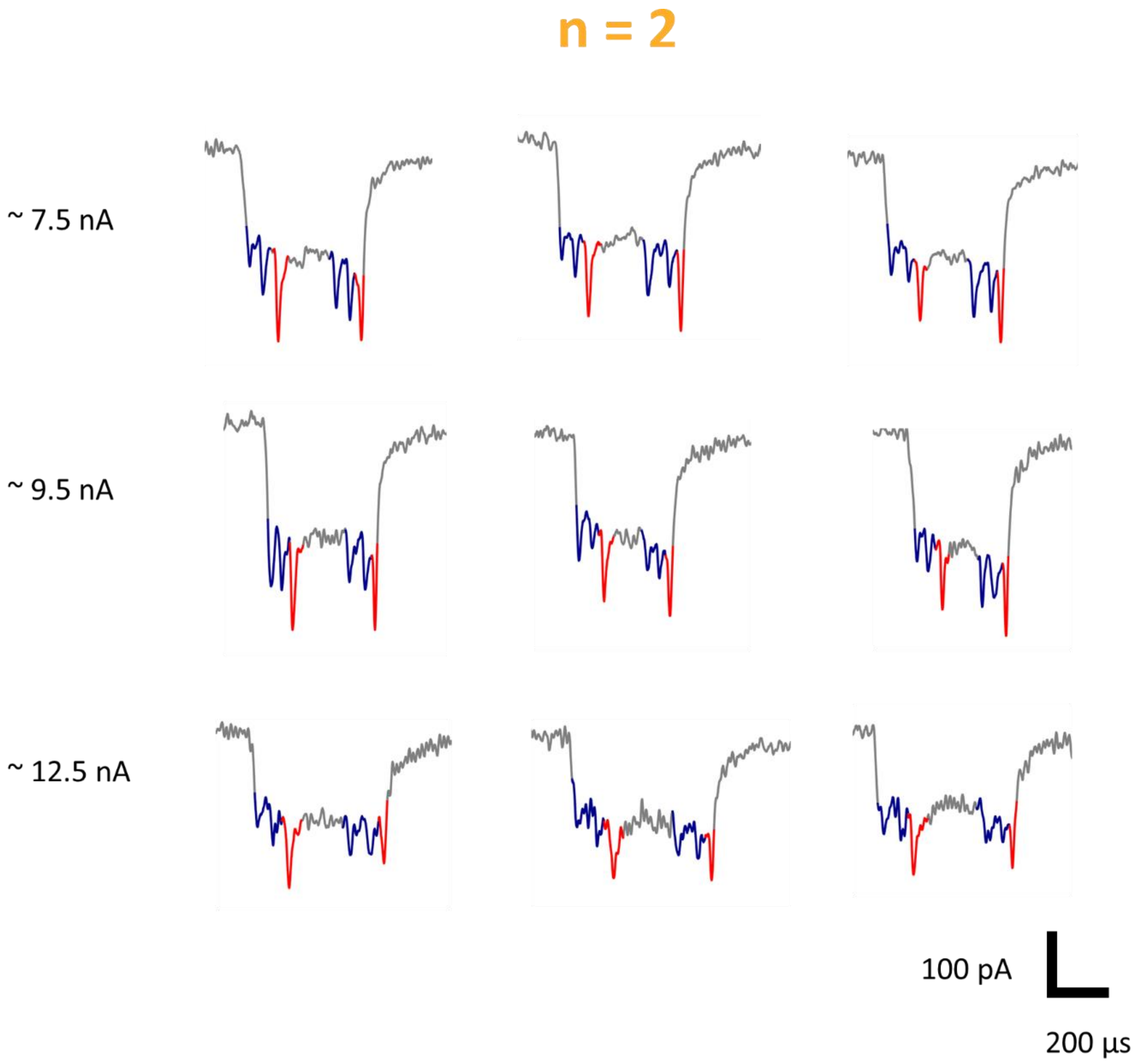

Supplementary Figure 13. N = 2 example events. Example nanopore events of RNA IDs produced from two transcription cycles (N = 2) measured in pores with different sizes, which exhibit different signal to noise ratio. Variation in pore size can be inferred from changes in the ionic current baseline. All measurements were performed under the same applied voltage of 600 mV. Larger pores have a larger ionic current baseline.

Supplementary Figure 14.

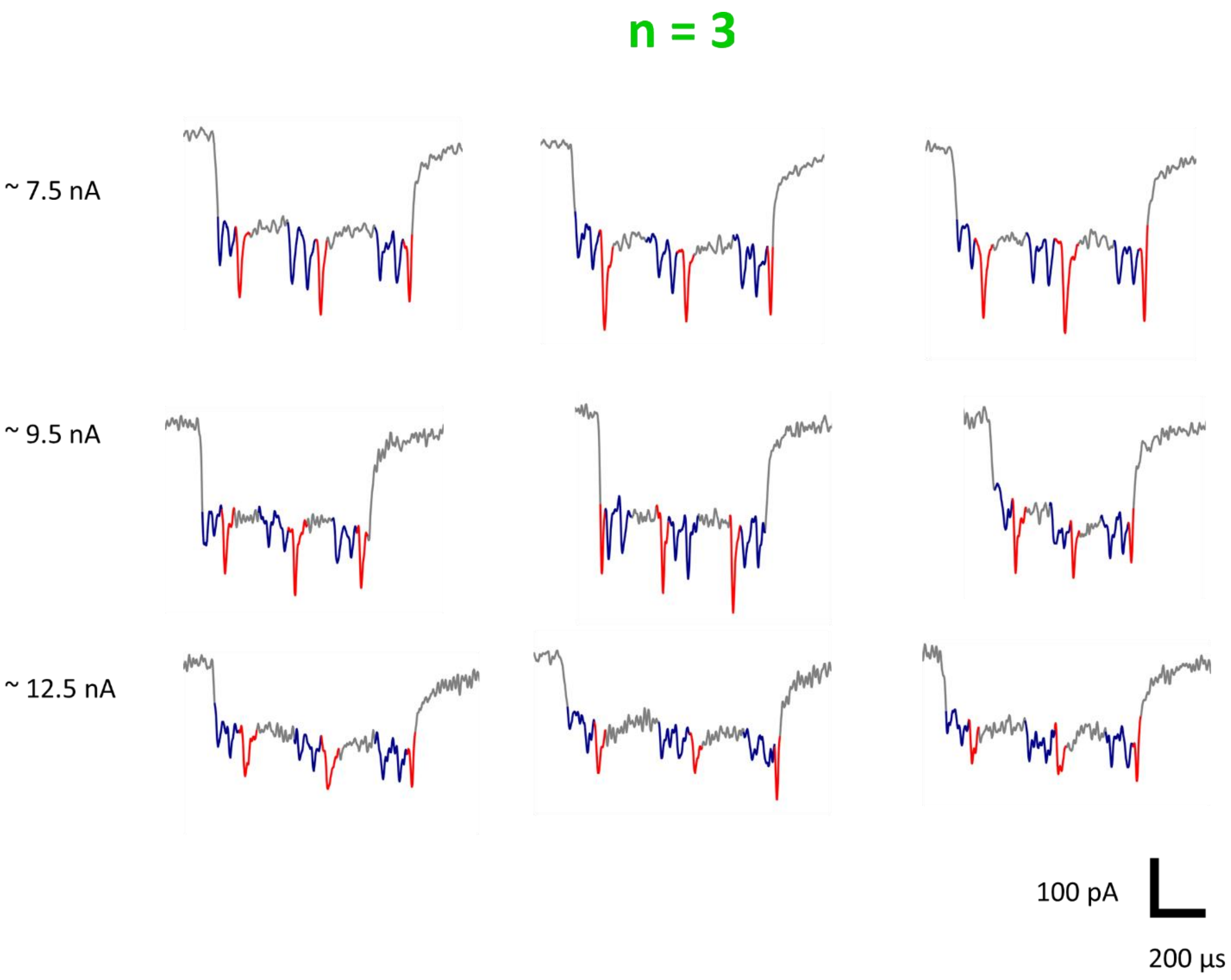

Supplementary Figure 14. N = 3 example events. Example nanopore events of RNA IDs produced from three transcription cycles (N = 3) measured in pores with different sizes, which exhibit different signal to noise ratio. Variation in pore size can be inferred from changes in the ionic current baseline. All measurements were performed under the same applied voltage of 600 mV. Larger pores have a larger ionic current baseline.

Supplementary Figure 15.

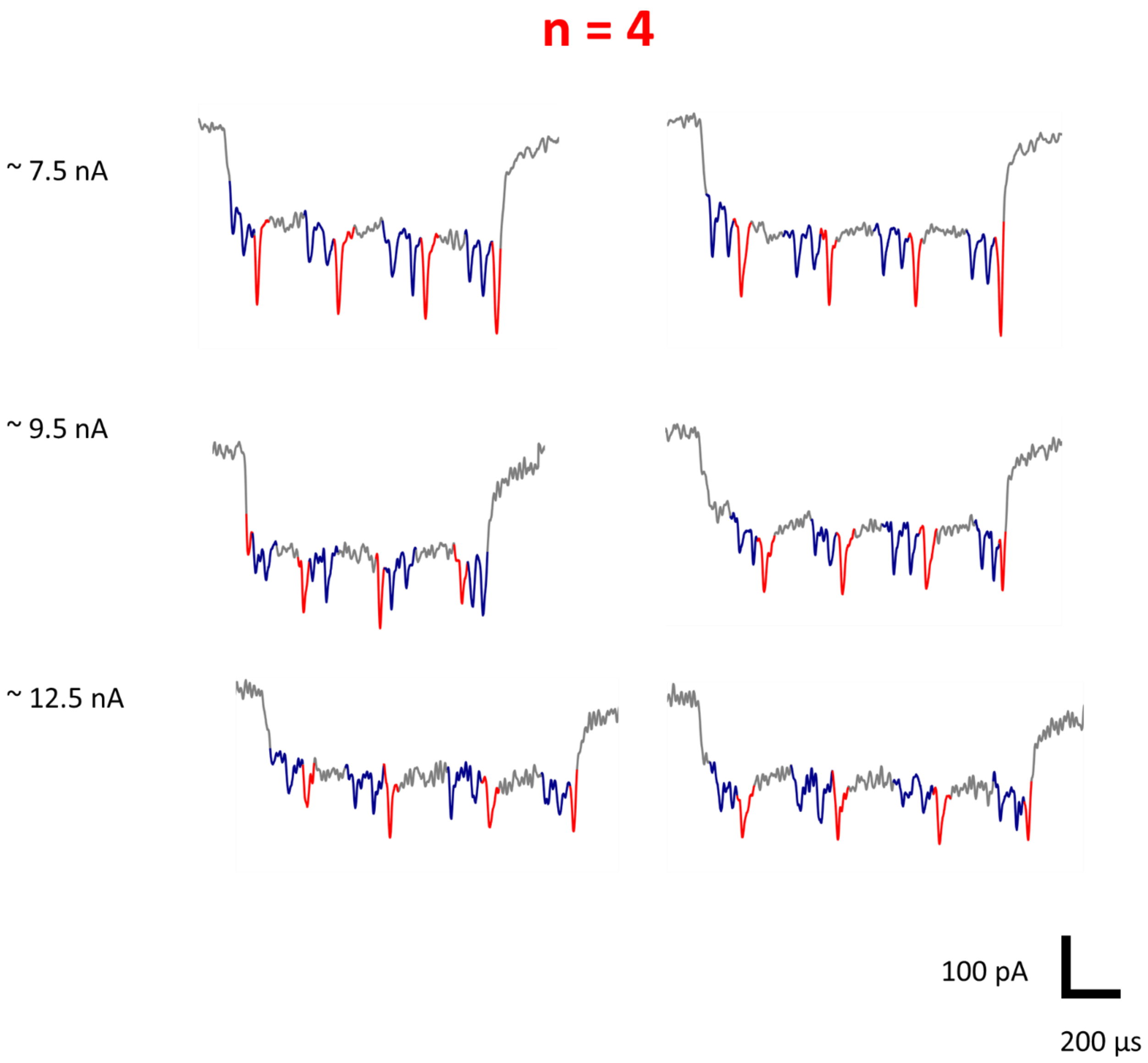

Supplementary Figure 15. N = 4 example events. Example nanopore events of RNA IDs produced from four transcription cycles (N = 4) measured in pores with different sizes, which exhibit different signal to noise ratio. Variation in pore size can be inferred from changes in the ionic current baseline. All measurements were performed under the same applied voltage of 600 mV. Larger pores have a larger ionic current baseline.

Supplementary Figure 16.

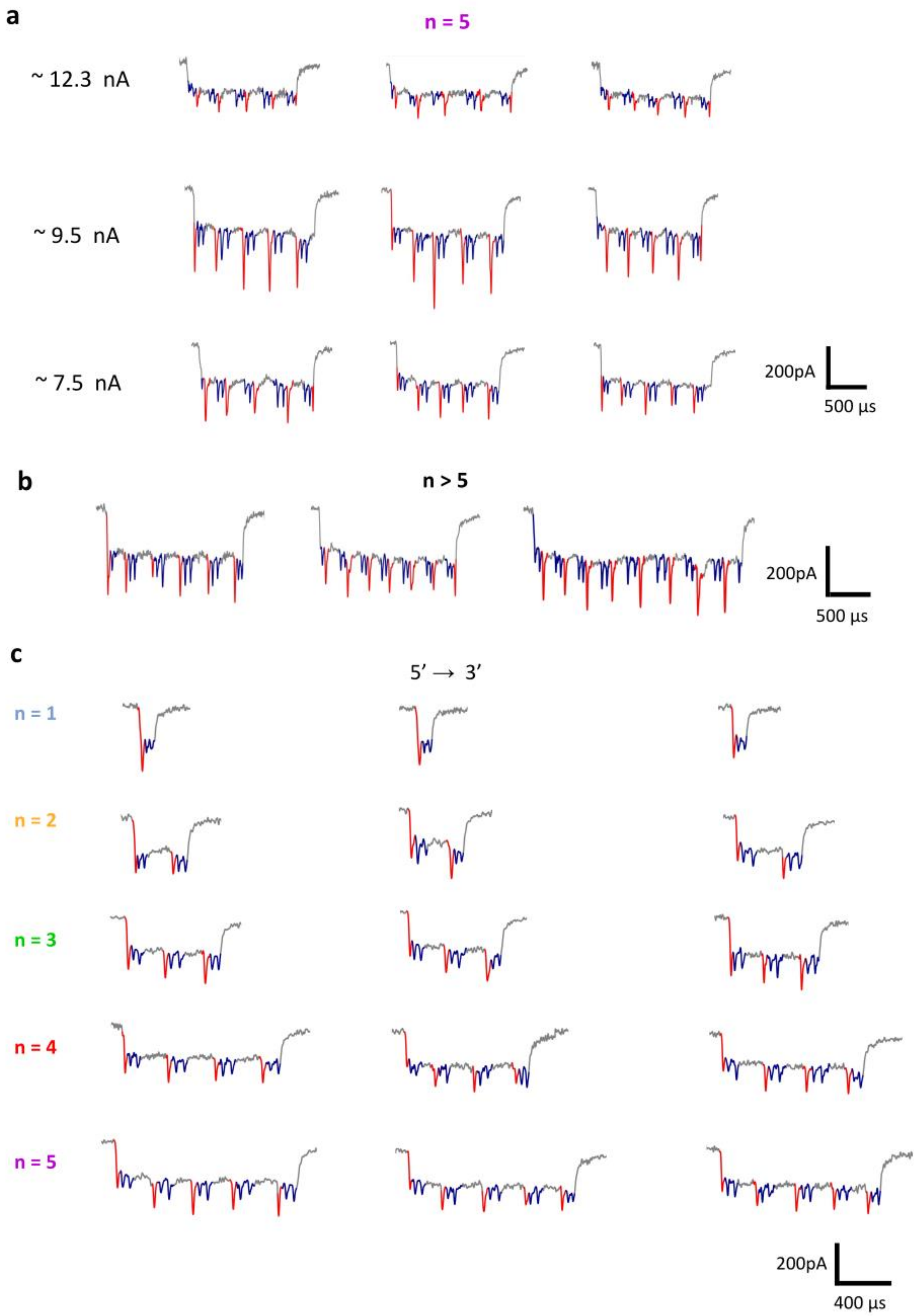

Supplementary Figure 16. RNA ID nanopore events for transcription cycles N = 5 and N > 5 example events. **a** Example of nanopore events of RNA IDs produced from five transcription cycles (N = 5) measured in pores with different sizes. All measurements were performed under the same applied voltage of 600 mV. **b** Also, RNA IDs with N > 5 were identified, example events are shown from different nanopore measurements. **c** Translocation of RNA IDs for each transcription cycle that entered to the pore in the 5′ to 3′ direction.

Supplementary Figure 17.

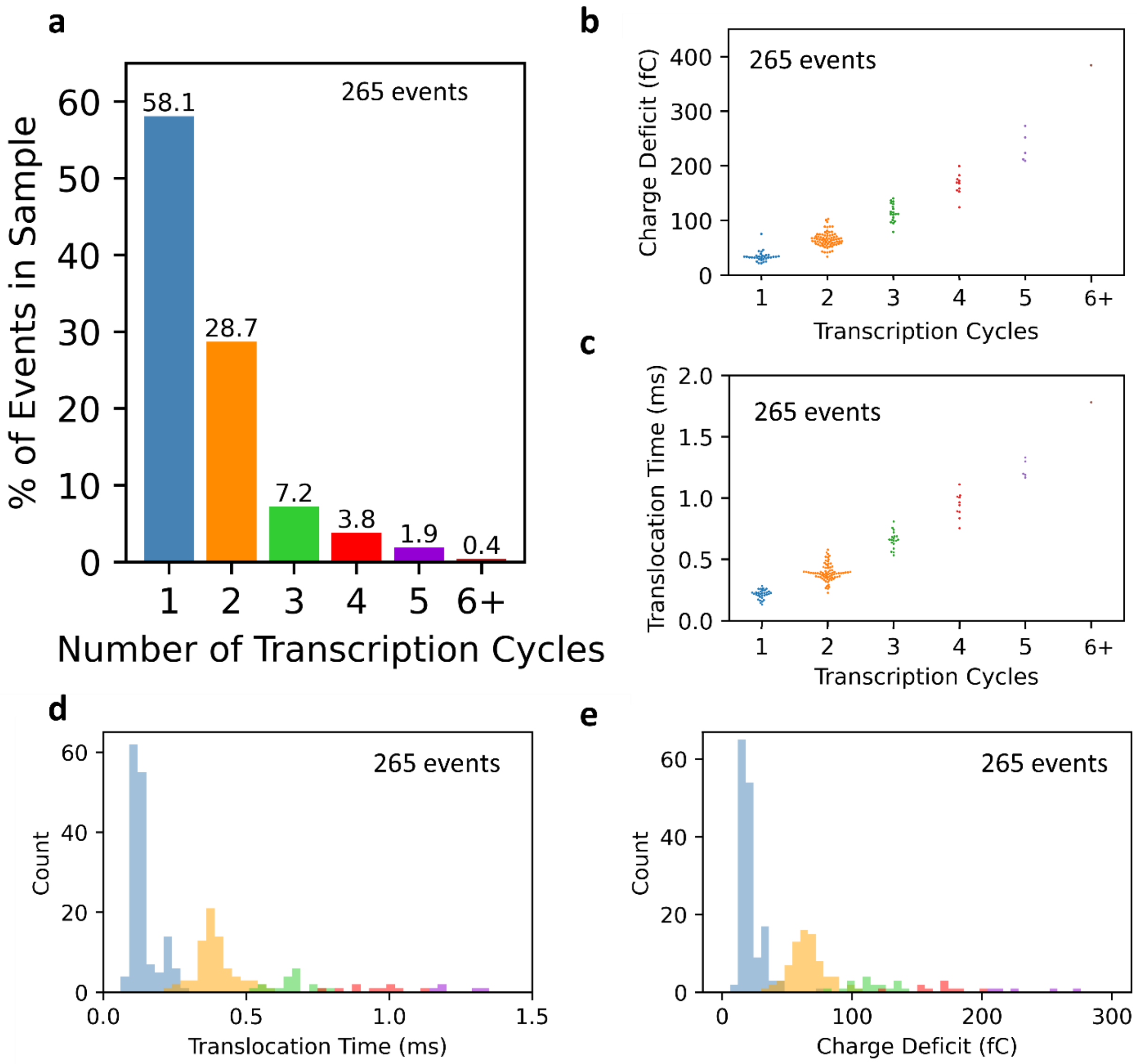

Supplementary Figure 17. Translocation time and charge deficit of RNA IDs from rolling circle transcription. **a** For individual nanopore measurements presented in Figure 3e, 265 unfolded events were identified. Bar plots show the relative abundance of each RNA ID classified by number of transcriptions cycle. **b** Swarm plot of charge deficit per transcription cycle. **c** Swarm plot of translocation time per transcription cycle. **c** and **d** correspond to histograms of translocation time and charge deficit, respectively, presented in Figure 3e, but using a linear scale on the *y* axis instead of a logarithmic scale.

Supplementary Figure 18.

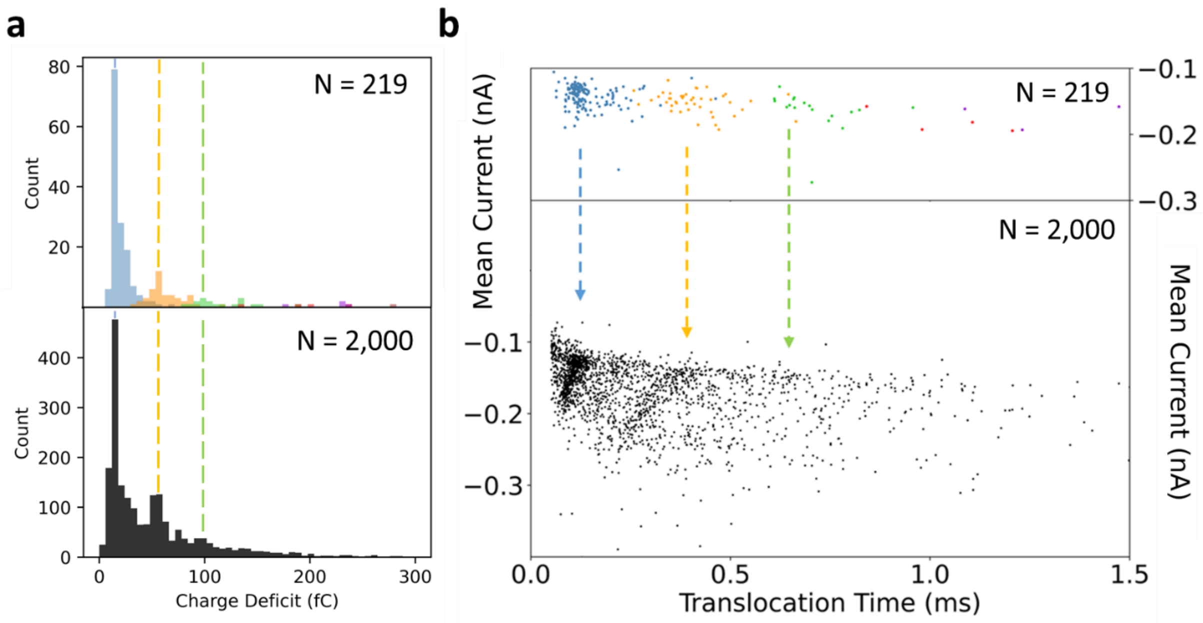

Supplementary Figure 18. Unfolded (linear) RNA IDs events are representative of the events with different conformations. **a** Histogram of charge deficit for all translocations detected in one nanopore measurement (black, 2000 events). The selection of 219 unfolded events shows a distribution of charge deficit which is representative of the distribution of all translocations detected, therefore these events can be used to describe the sample and gain single-molecule information from the RNA ID design. The charge density distributions produced between the main distributions (green and yellow) are ascribed to fall-off of T7RNAP. **b** Scatter plot of mean current against translocation time, which also shows that the selection of unfolded can be used for the description of a sample within a defined parameter space.

Supplementary Figure 19.

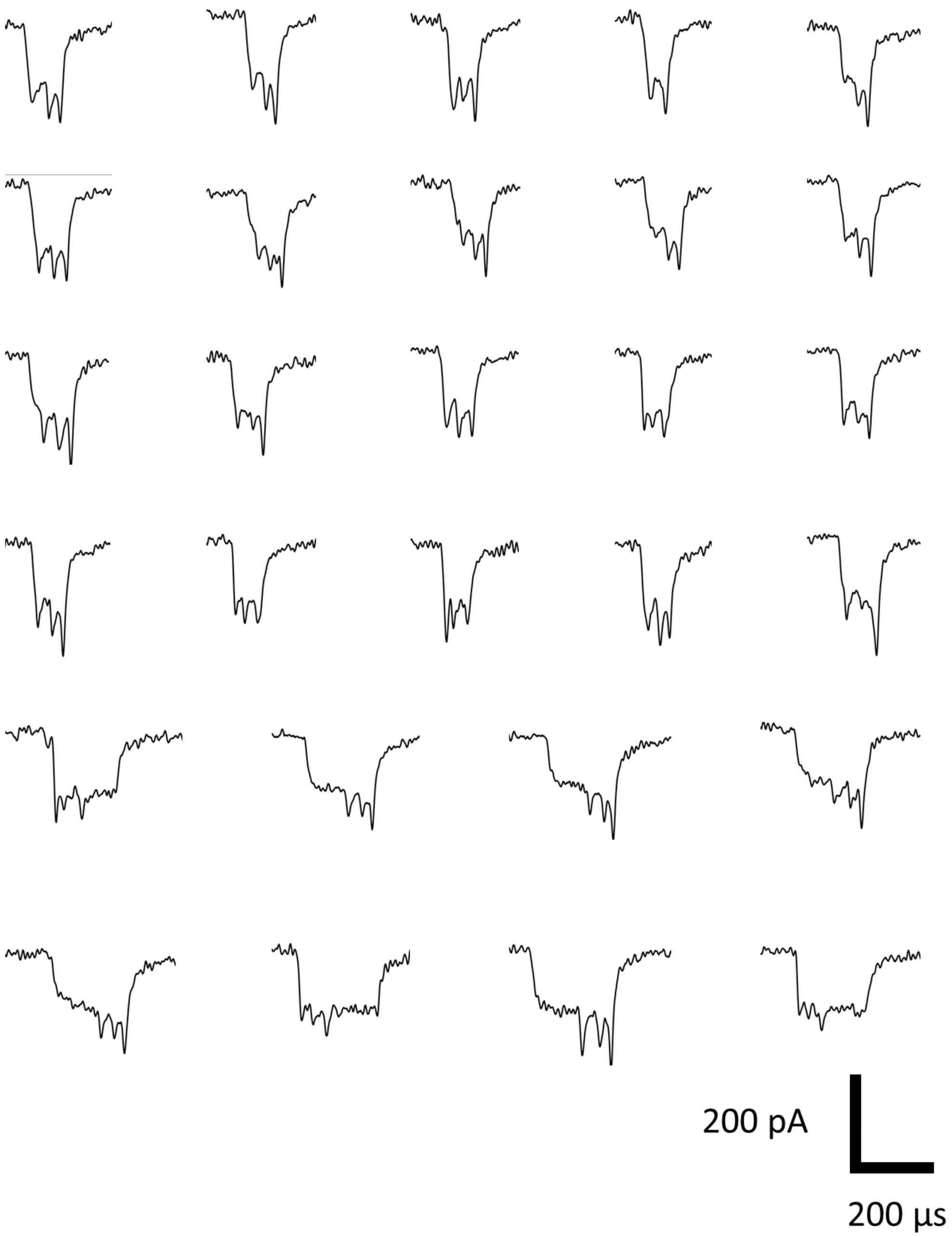

Supplementary Figure 19. Example nanopore events of RNA IDs produced from one transcription cycle (N = 1) used for single-molecule sizing in Figure 4d. These are the first 28 unfolded translocation events detected for N = 1.

Supplementary Figure 20.

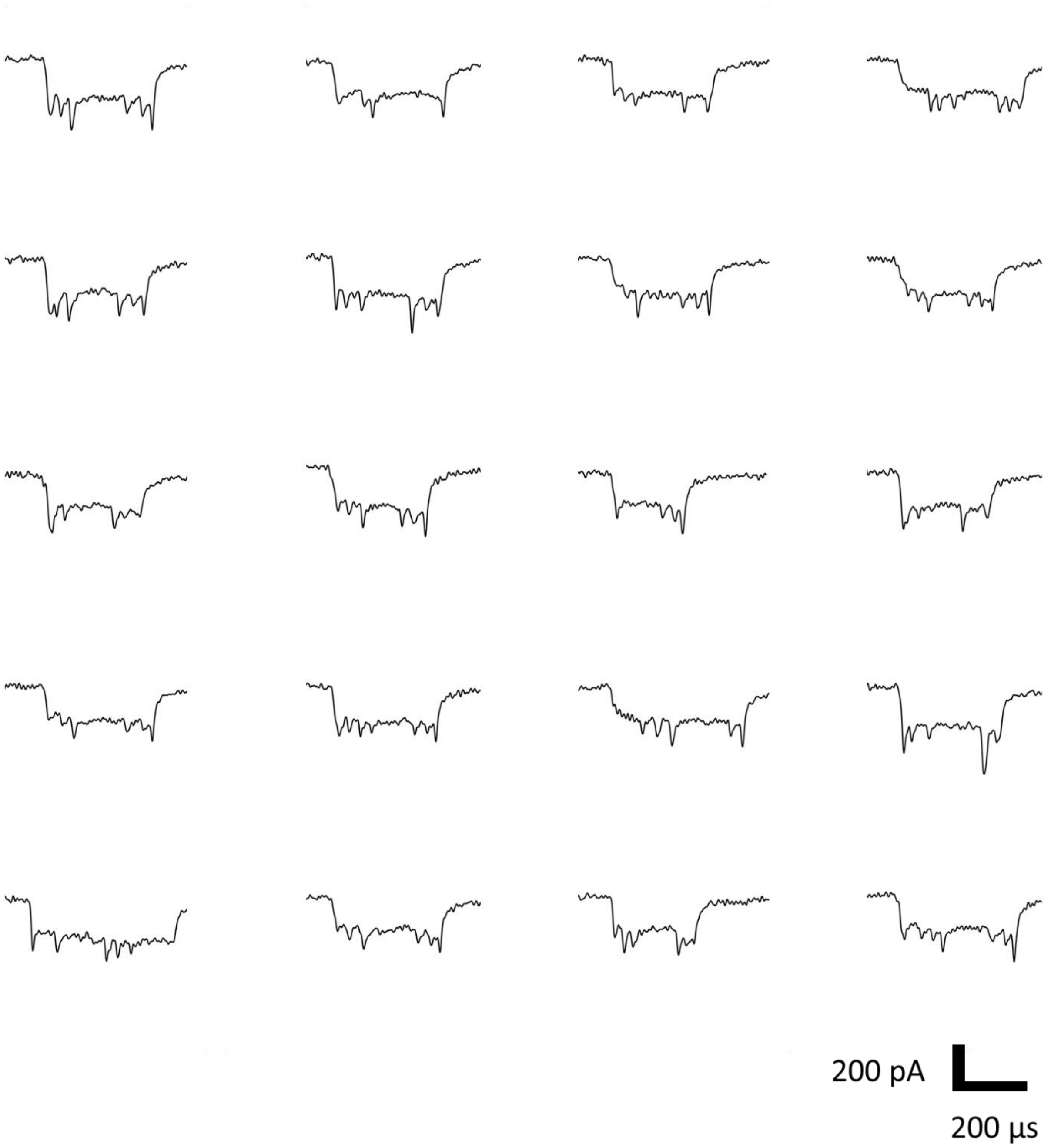

Supplementary Figure 20. Example nanopore events of RNA IDs produced from two transcription cycles (N = 2) used for single-molecule sizing in Figure 4d. These are the first 20 unfolded translocation events detected for RNA IDs with N = 2.

Supplementary Figure 21.

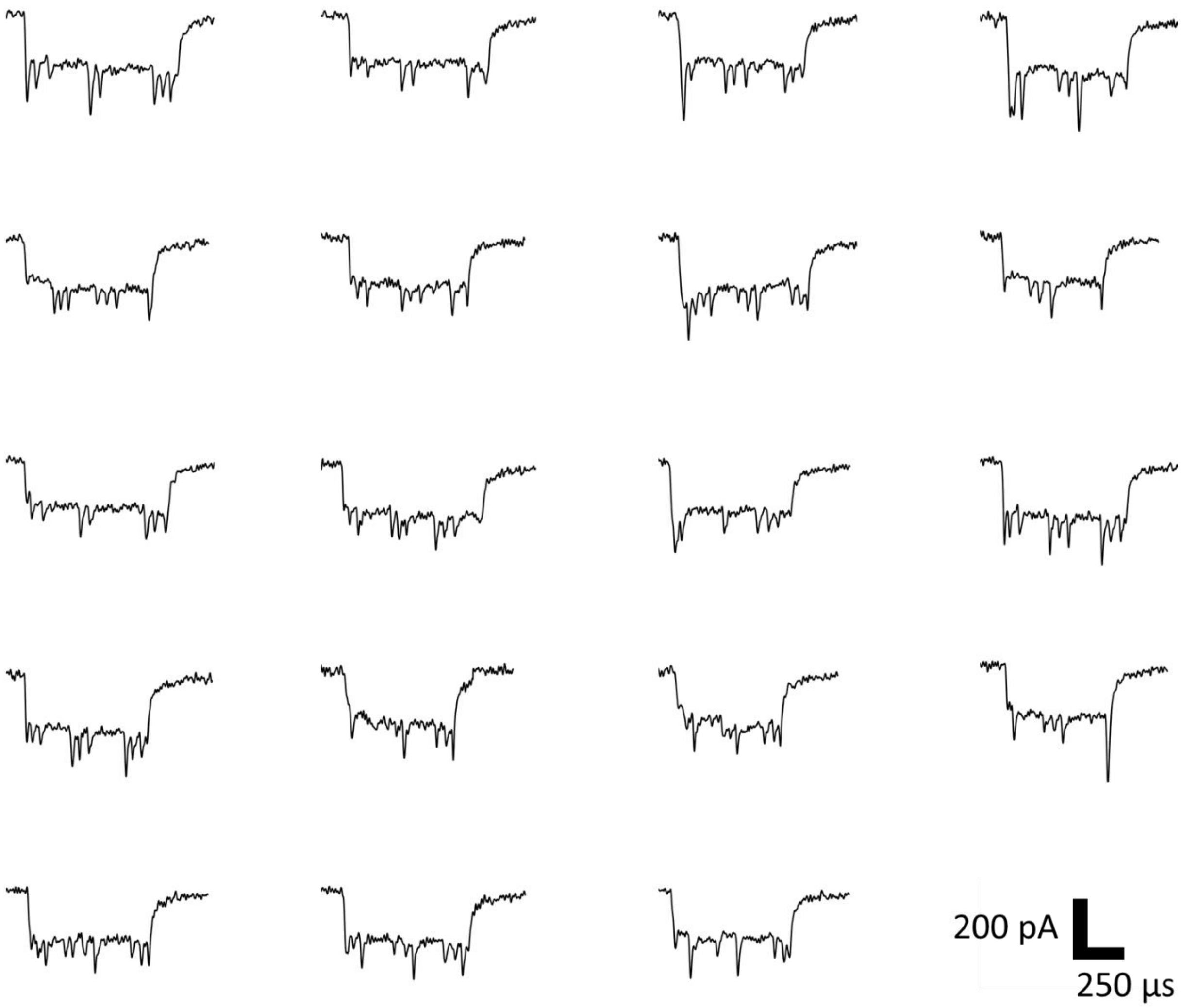

Supplementary Figure 21. Example nanopore events of RNA IDs produced from three transcription cycles (N = 3) used for single-molecule sizing in Figure 4d. These are the first 20 unfolded translocation events detected for RNA IDs with N = 3.

Supplementary Figure 22.

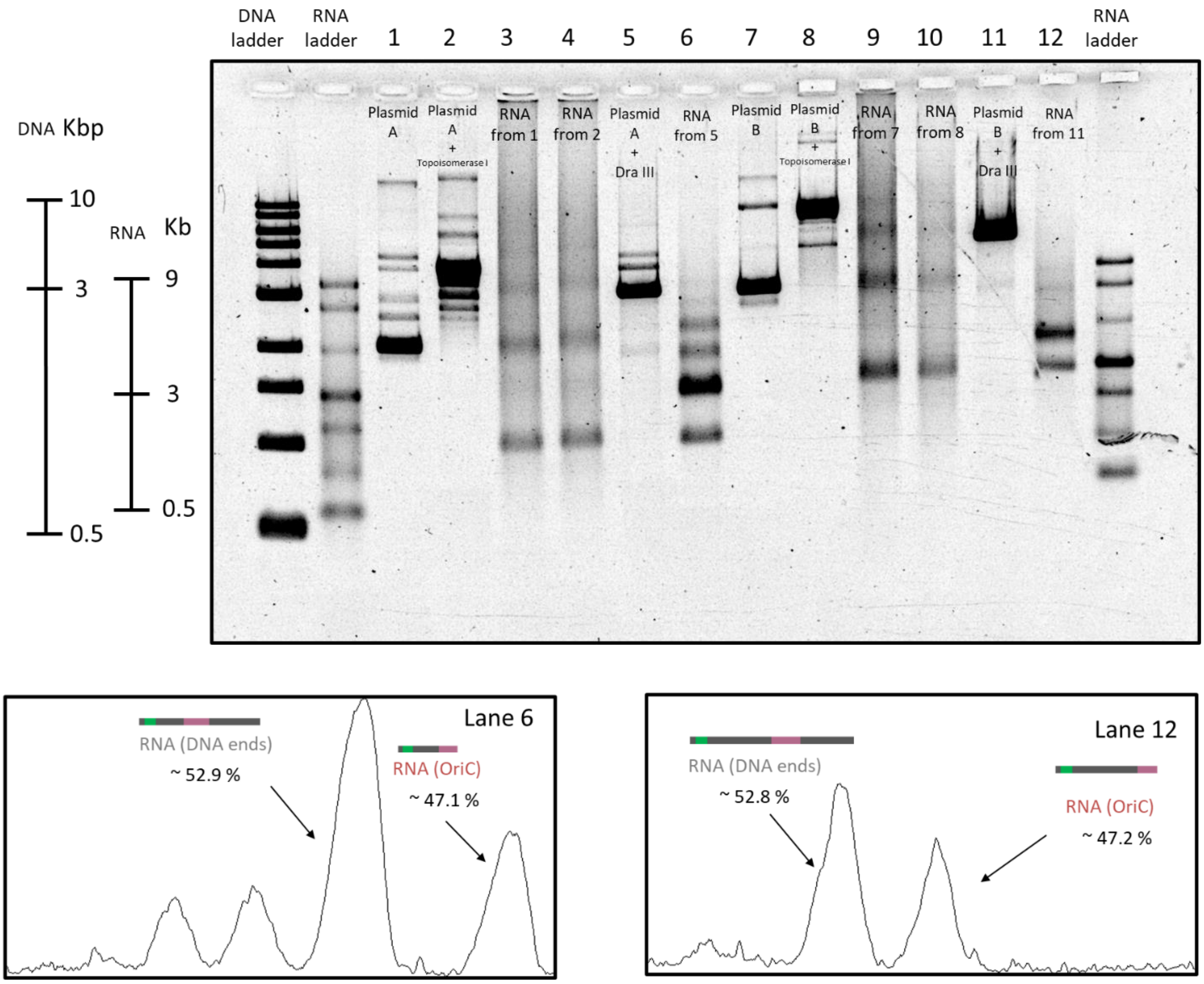

Plasmid	Molecule	Intensity profile (a.u.)	Normalized intensity profile (a.u./bp)
**A**	RNA (OriC)	8181.711	5.389
	RNA (Template end)	17336.317	6.049
**B**	RNA (OriC)	5889.418	1.983
	RNA (Template end)	9616.418	2.221

Supplementary Figure 22. Transcription of circular and linear DNA templates with the OriC at different distances from the T7 RNA polymerase promoter. DNA ladder and ssRNA ladder are included. Lane 1 – circular DNA plasmid with 12 CTG repeats (sequence in Supplementary Table 1). Lane 2 – circular plasmid from lane 1 treated with *Escherichia coli* Topoisomerase I. Lane 3 – DNase I treated RNA from transcription of circular plasmid in lane 1. Lane 4 – DNase I treated RNA from transcription of Topoisomerase I treated circular plasmid in lane 2.

Lane 5 – linear DNA digested using DraIII-HF. Lane 6 – DNase treated RNA from transcription of linear DNA in lane 5. Lane 7 – 4527 kbp circular DNA plasmid (sequence in Supplementary Table 4). Lane 8 – circular plasmid from lane 7 treated with *Escherichia coli* Topoisomerase I. Lane 9 – DNase I treated RNA from transcription of circular plasmid in lane 7. Lane 10 – DNase I treated RNA from transcription of Topoisomerase I treated circular plasmid in lane 8. Lane 11 – linear DNA digested using DraIII-HF, it is a longer linear DNA than the linear template in lane 5. Lane 12 – DNase treated RNA from transcription of linear DNA in lane 11. The topmost band of lane 12, slightly below 5 kb, corresponds to transcription of the entire linear template, while the band from the bottom, allocated slightly below 3 kb, corresponds to premature termination in the OriC. The position of the bands is in good agreement with the expected premature termination in the OriC (see sequence in Supplementary Table 4). Gel: 1 % (w/v) agarose, 1 × TBE, 0.02% sodium hypochlorite. The intensity profile of both bands in lanes 6 and 12 were plotted and the area of each peak was computed. The peak areas were normalized by the number of base pairs of each transcript to obtain an estimate of transcript abundance. Gel suggests premature transcription termination of ~ 47 % in the OriC in both cases.

Supplementary Figure 23.

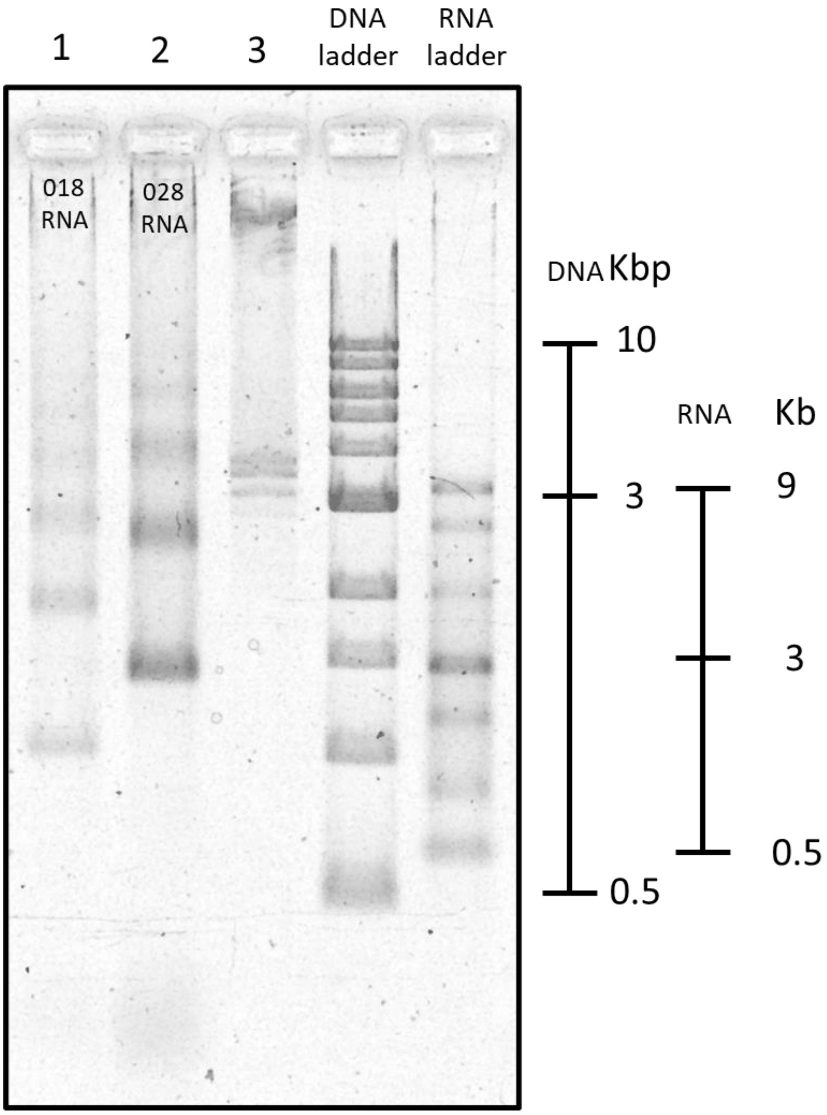

Supplementary Figure 23. Rolling circle transcription of circular DNA constructs with different sizes and different distances between the OriC and T7RNAP promoter. The agarose gel shows a DNA ladder and ssRNA ladder on the right side. Lane -1 shows RNA product from rolling circle transcription of circular DNA construct (sequence in Supplementary Table 1) as presented in Figure 3d. Lane 2 displays RNA product from transcription of a larger DNA construct (sequence in Supplementary Table 4) with a larger separation between the OriC and promoter (~3kbp). Each band is ascribed to transcription termination at the OriC at the different transcription cycles. The Content of lane 3 is unknown. Gel: 1 % (w/v) agarose, 1 × TBE, 0.02% sodium hypochlorite.

Supplementary Figure 24.

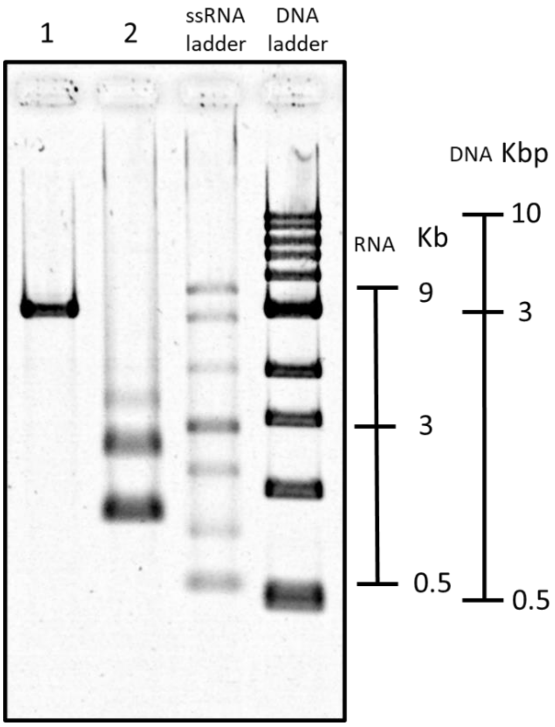

Supplementary Figure 24. Transcription of pFGG linear DNA template. ssRNA and DNA ladder are included on the right side of the gel. Lane 1 – 2972 kbp DNA construct with no tandem repeats (sequence in Supplementary Table 5) linearized with ScaI. Lane 2 – DNase treated RNA from transcription of linear DNA in lane 1. The bottom band is ascribed to transcription until the restriction site of ScaI (1192 bp), where termination occurs as the T7RNAP falls off from the DNA template. The top band, located between 2 and 3 kb corresponds to termination at the OriC in uncut plasmids. Gel: 1 % (w/v) agarose, 1 × TBE, 0.02% sodium hypochlorite.

Supplementary Figure 25.

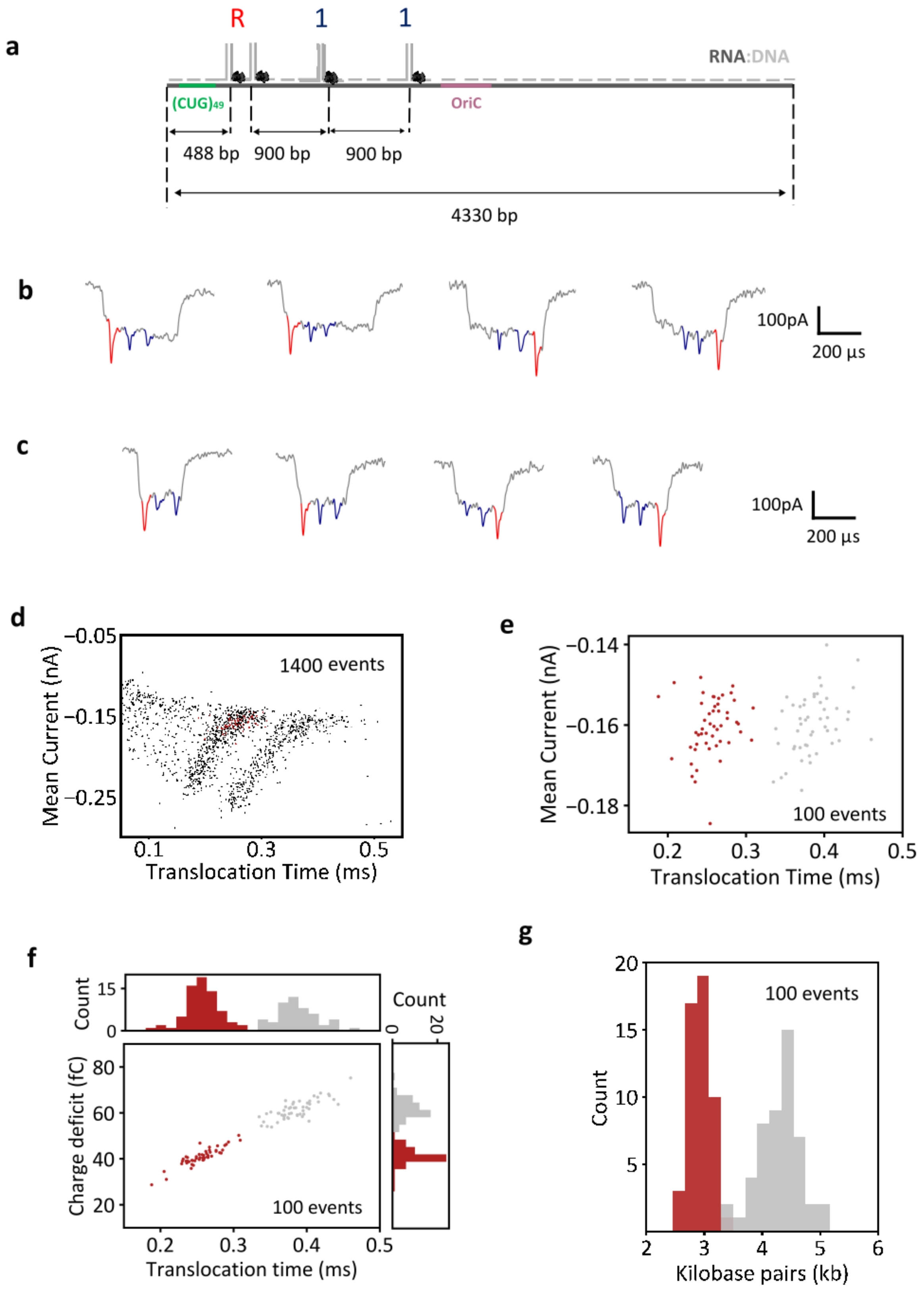

Supplementary Figure 25: Single molecule sizing of RNA IDs produced from a 4.5 kbp DNA construct. **a** The sequence of the DNA construct is presented in Supplementary Table 4. The linearized version of the construct (DraIII) is presented Supplementary Figure 22, in lane 11, and the transcription products are shown in lane 12. The RNA ID design includes an ‘R’ label and two ‘1’ bits (Supplementary Table 6). The oligos used for assembly of the hybrid are shown in supplementary Table 6. **b** Exemplary RNA ID translocation events of full-length transcripts (END). **c** Exemplary RNA ID translocation events ascribed to premature termination (PT). **d** Scatter plot of mean current against translocation time shows two distinct distributions, attributed to PT RNA IDs and END RNA IDs (from left to right). **e** Scatter plot of mean current against translocation time of 100 unfolded events. **f** Scatter plot of charge deficit against translocation time for RNA IDs of PT (red) and END (gray) RNA transcripts, which shows the linear dependence of both parameters. **g** Base pair length of molecules converted from translocation time (in f), which shows two distinct distributions. PT distribution has a mean length of (2.9 ± 0.2) kbp and END transcripts have a mean length of (4.3 ± 0.3) kbp. Errors correspond to standard deviation.

Supplementary Figure 26.

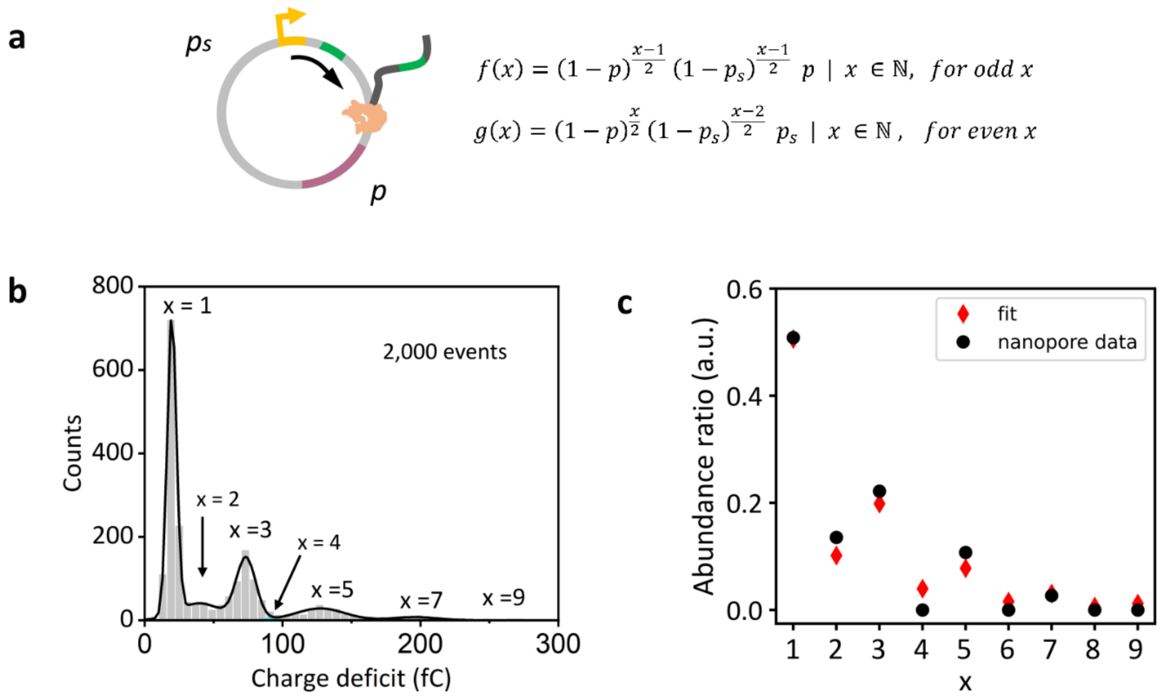

Supplementary Figure 26: Quantitative description of T7RNAP processivity and transcription termination. **a** Considering a probability *p* of transcription terminating at the identified premature transcription termination site, and a probability p_s for transcription terminating solely by dissociation of the polymerase at a different region of the plasmid. Equation *f(x)* describes the abundance of transcripts originated from premature transcription termination at OriC. Equation *g(x)* describes the transcript abundance originated from fall-off of T7RNAP at a different region of the plasmid. **b** The distribution of the charge deficit of nanopore translocation events is presented. The different transcript populations were fitted to gaussian functions, from which the relative abundance of transcripts was derived. The distributions ascribed to premature termination transcripts are labelled with *x* values of odd integers (*x* = 1, 3, 5, 7) and the minor distributions ascribed to transcripts produced from T7RNAP dissociation in a different region of the plasmid receive *x* values of even integers (*x* = 2, 4, 6, 8). **c** The relative abundance of transcripts (black) are plotted. Fitting of *f(x)* and *g(x)* is plotted in red, $p \sim 0.51$ and $p_s \sim 0.21$, which agrees with transcription termination reported in linear DNA constructs.

Supplementary Figure 27.

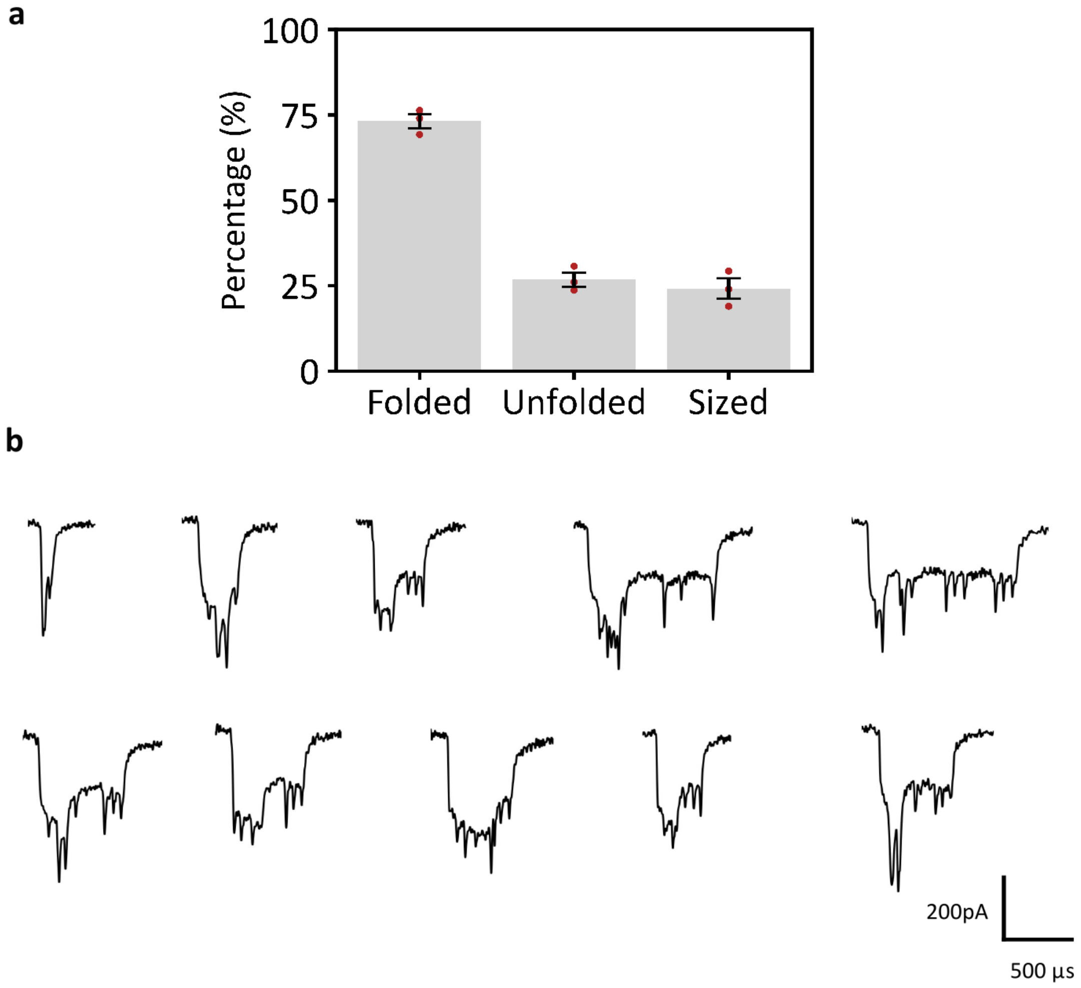

Supplementary Figure 27: Percentage of translocation events sized. **a** Shows the percentage of folded, unfolded and sized events. The percentages were computed from 3 different nanopore measurements of RNA IDs produced from transcription of a circular DNA construct. The amount of folded events correspond to (73 ± 2)%, unfolded events constitute (27 ± 2)% of the sample, and (24 ± 3)% were sized. The errors correspond to the standard error of the mean. **b** Exemplary folded events are presented.

Supplementary Figure 28

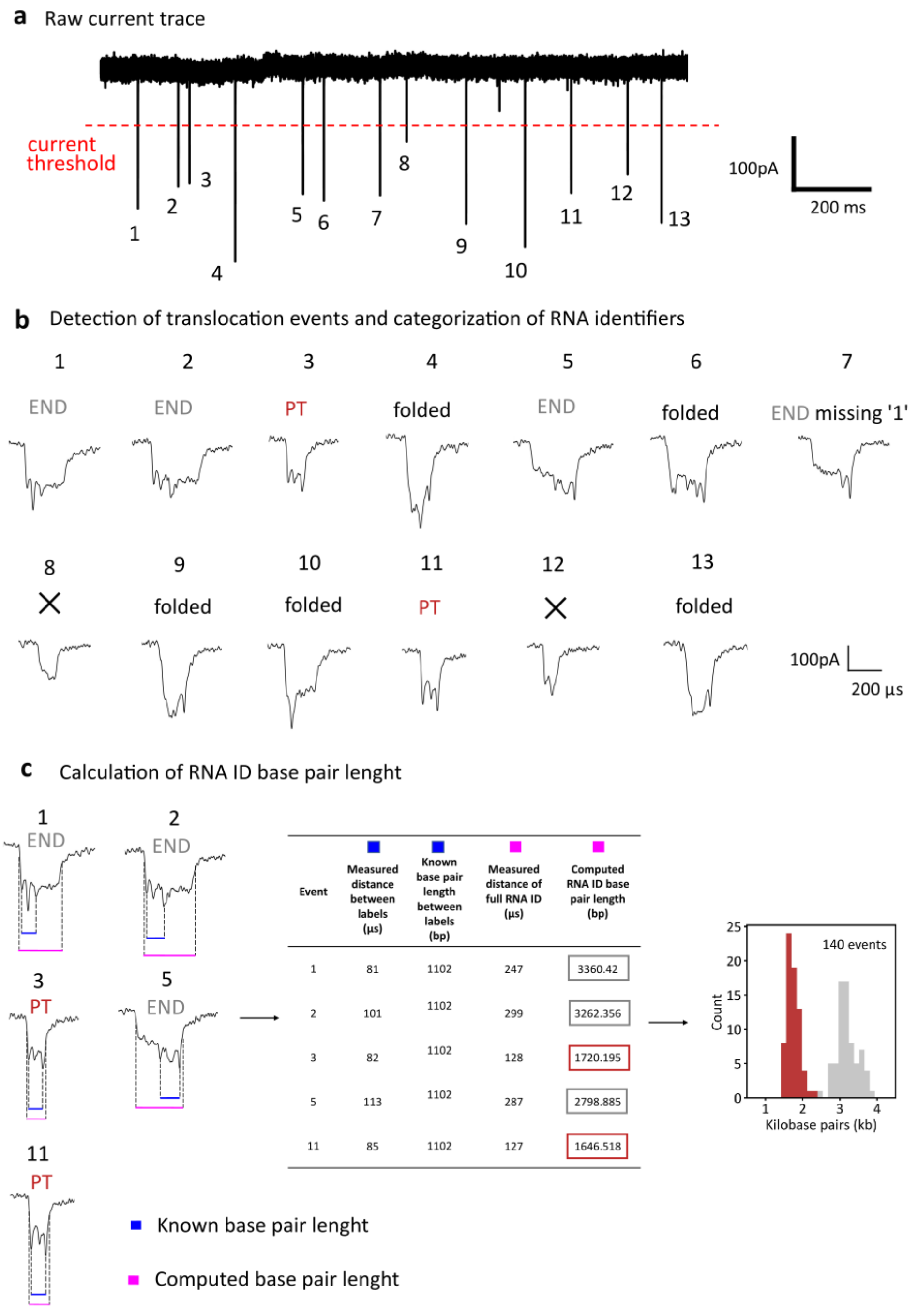

Supplementary Figure 28: Step-by-step nanopore data analysis for RNA ID sizing. **a** An exemplary region of the ionic raw current trace is shown. We use a home-built LabVIEW code to identify translocation events by simple thresholding. Events are then analysed by using standard parameters, which include the charge deficit, minimum translocation time and mean current that are given by the length of the RNA ID molecule and its design. **b** The translocation events were then categorized into PT RNA ID, END RNA ID, folded RNA ID and

translocations associated to misfolded molecules (marked X) based on the ionic current trace of the events. **c** The base pair length of PT RNA IDs and END RNA IDs was computed using the RNA ID design. The distance between current spikes (blue) was measured (in time units). This distance corresponds to a known base pair length which was used to calculate a conversion factor. Then, the length in time units of the entire translocation event was measured (pink) and converted into a base. The base pair length was plotted as shown in the right.

Supplementary Figure 29

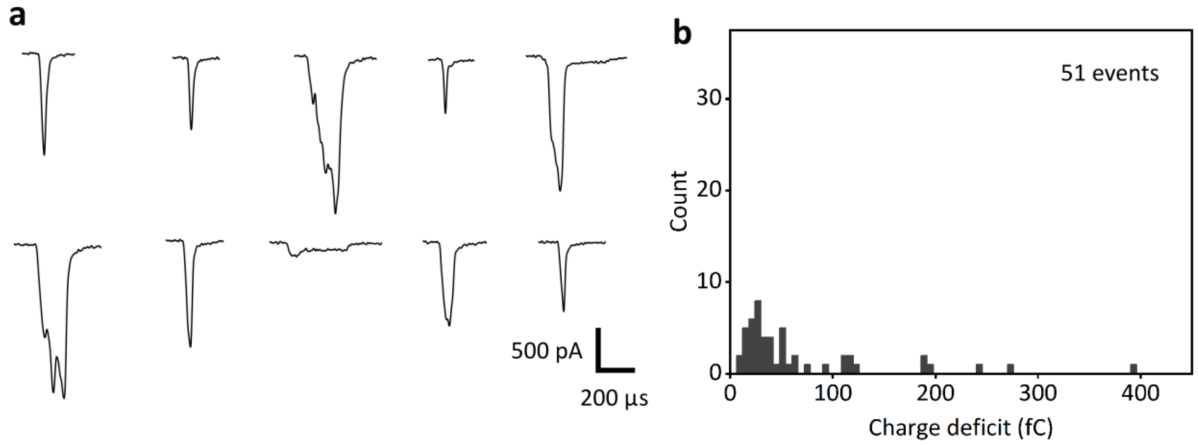

Supplementary Figure 29: Characterization of ionic current trace shows that distinctive current traces emanate exclusively from the RNA IDs. **a** A nanopore measurement was performed in the absence of RNA IDs for 90 minutes. Translocation events detected using the following threshold parameters are shown. Minimum charge deficit: 0 fC, maximum charge deficit 400 fC, minimum translocation time: 50 µs, minimum current drop: -100 pA. Ionic current scale bar corresponds to 500 pA, and translocation time scale bar to 200 µs. **b** The charge deficit of the translocations detected are plotted, demonstrating that the current traces studied in this work originate from the translocation of RNA IDs.

Supplementary Table 1.

Sequence of modified pJET1.2/blunt cloning vector (CloneJET PCR Cloning Kit, Thermo Fisher, Catalog number: K1231): Circular DNA (3062 bp) with inserted $(CTG)_{12}$ tandem repeats. Map of the plasmid is included at the end of the table.

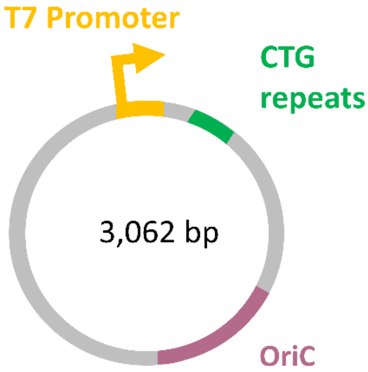

Table 1

Sequence (5′→ 3′)
T7 promoter $(CTG)_{12}$ repeats   OriC   DraIII Cutting
GCCCCTGCAGCCGAATTATATTATTTTTGCCAAATAATTTTTAACAAAAGCTCTGAAGTCTTCTTC<br>ATTTAAATTCTTAGATGATACTTCATCTGGAAAATTGTCCCAATTAGTAGCAT**CACGCTGTG**AGTA<br>AGTTCTAAACCATTTTTTTATTGTTGTATTATCTCTAATCTTACTACTCGATGAGTTTTCGGTATT<br>ATCTCTATTTTTAACTTGGAGCAGGTTCCATTCATTGTTTTTTTCATCATAGTGAATAAAATCAAC<br>TGCTTTAACACTTGTGCCTGAACACCATATCCATCCGGCG**TAATACGACTCACTATAGG**GAGAGCG<br>GCCGCCAGATCTTCCGGATGGCTCGAGTTTTTCAGCAAGATCATGGTGCAGTGTAGCCGGGAATG**C**<br>**TGCTGCTGCTGCTGCTGCTGCTGCTGCTGCTGCTG**GGGGGATCACAGACCATTCTCGGCTTAAATC<br>TTTCTAGAAGATCTCCTACAATATTCTCAGCTGCCATGGAAAATCGATGTTCTTCTTTTATTCTCT<br>CAAGATTTTCAGGCTGTATATTAAAACTTATATTAAGAACTATGCTAACCACCTCATCAGGAACCG<br>TTGTAGGTGGCGTGGGTTTTCTTGGCAATCGACTCTCATGAAAACTACGAGCTAAATATTCAATAT<br>GTTCCTCTTGACCAACTTTATTCTGCATTTTTTTTGAACGAGGTTTAGAGCAAGCTTCAGGAAACT<br>GAGACAGGAATTTTATTAAAAATTTAAATTTTGAAGAAAGTTCAGGGTTAATAGCATCCATTTTTT<br>GCTTTGCAAGTTCCTCAGCATTCTTAACAAAAGACGTCTCTTTTGACATGTTTAAAGTTTAAACCT<br>CCTGTGTGAAATTATTATCCGCTCATAATTCCACACATTATACGAGCCGGAAGCATAAAGTGTAAA<br>GCCTGGGGTGCCTAATGAGTGAGCTAACTCACATTAATTGCGTTGCGCTCACTGCCAATTGCTTTC<br>CAGTCGGGAAACCTGTCGTGCCAGCTGCATTAATGAATCGGCCAACGCGCGGGGAGAGGCGGTTTG<br>CGTATTGGGCGCTCTTCCGCTTCCTCGCTCACTGACTCGCTGCGCTCGGTCGTTCGGCTGCGGCGA<br>GCGGTATCAGCTCACTCAAAGGCGGTAATACGGTTATCCACAGAATCAGGGGATAACGCAGGAAAG<br>AACATGTGAGCAAAAGGCCAGCAAAAGGCCAGGAACCGTAAAAAGGCCGCGTTGCTGGCGTT**TTTC**<br>**CATAGGCTCCGCCCCCCTGACGAGCATCACAAAAATCGACGCTCAAGTCAGAGGTGGCGAAACCCG**<br>**ACAGGACTATAAAGATACCAGGCGTTTCCCCCTGGAAGCTCCCTCGTGCGCTCTCCTGTTCCGACC**<br>**CTGCCGCTTACCGGATACCTGTCCGCCTTTCTCCCTTCGGGAAGCGTGGCGCTTTCTCATAGCTCA**

CGCTGTAGGTATCTCAGTTCGGTGTAGGTCGTTCGCTCCAAGCTGGGCTGTGTGCACGAACCCCCC
GTTCAGCCCGACCGCTGCGCCTTATCCGGTAACTATCGTCTTGAGTCCAACCCGGTAAGACACGAC
TTATCGCCACTGGCAGCAGCCACTGGTAACAGGATTAGCAGAGCGAGGTATGTAGGCGGTGCTACA
GAGTTCTTGAAGTGGTGGCCTAACTACGGCTACACTAGAAGGACAGTATTTGGTATCTGCGCTCTG
CTGAAGCCAGTTACCTTCGGAAAAAGAGTTGGTAGCTCTTGATCCGGCAAACAAACCACCGCTGGT
AGCGGTGGTTTTTTTGTTTGCAAGCAGCAGATTACGCGCAGAAAAAAAGGATCTCAAGAAGATCCT
TTGATCTTTTCTACGGGGTCTGACGCTCAGTGGAACGAAAACTCACGTTAAGGGATTTTGGTCATG
AGATTATCAAAAAGGATCTTCACCTAGATCCTTTTAAATTAAAAATGAAGTTTTAAATCAATCTAA
AGTATATATGAGTAAACTTGGTCTGACAGTTACCAATGCTTAATCAGTGAGGCACCTATCTCAGCG
ATCTGTCTATTTCGTTCATCCATAGTTGCCTGACTCCCCGTCGTGTAGATAACTACGATACGGGAG
GGCTTACCATCTGGCCCCAGTGCTGCAATGATACCGCGAGACCCACGCTCACCGGCTCCAGATTTA
TCAGCAATAAACCAGCCAGCCGGAAGGGCCGAGCGCAGAAGTGGTCCTGCAACTTTATCCGCCTCC
ATCCAGTCTATTAATTGTTGCCGGGAAGCTAGAGTAAGTAGTTCGCCAGTTAATAGTTTGCGCAAC
GTTGTTGCCATTGCTACAGGCATCGTGGTGTCACGCTCGTCGTTTGGTATGGCTTCATTCAGCTCC
GGTTCCCAACGATCAAGGCGAGTTACATGATCCCCCATGTTGTGCAAAAAAGCGGTTAGCTCCTTC
GGTCCTCCGATCGTTGTCAGAAGTAAGTTGGCCGCAGTGTTATCACTCATGGTTATGGCAGCACTG
CATAATTCTCTTACTGTCATGCCATCCGTAAGATGCTTTTCTGTGACTGGTGAGTACTCAACCAAG
TCATTCTGAGAATAGTGTATGCGGCGACCGAGTTGCTCTTGCCCGGCGTCAATACGGGATAATACC
GCGCCACATAGCAGAACTTTAAAAGTGCTCATCATTGGAAAACGTTCTTCGGGGCGAAAACTCTCA
AGGATCTTACCGCTGTTGAGATCCAGTTCGATGTAACCCACTCGTGCACCCAACTGATCTTCAGCA
TCTTTTACTTTCACCAGCGTTTCTGGGTGAGCAAAAACAGGAAGGCAAAATGCCGCAAAAAAGGGA
ATAAGGGCGACACGGAAATGTTGAATACTCATACTCTTCCTTTTTCAATATTATTGAAGCATTTAT
CAGGGTTATTGTCTCATGAGCGGATACATATTTGAATGTATTTAGAAAAATAAACAAATAGGGGTT
CCGCGCACATTTCCCCGAAAAGTGCCACCTGACGTCTAAGAAACCATTATTATCATGACATTAACC
TATAAAAATAGGCGTATCACGAGGCC

Map of the plasmid showing restriction sites, T7RNAP promoter sequence, the OriC and the CTG repeats.

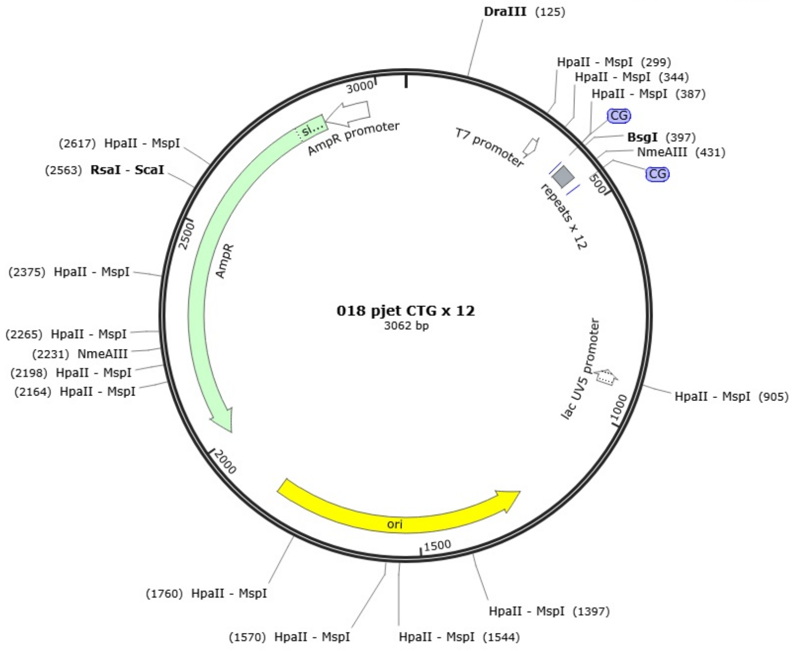

Supplementary Table 2.

Sequence of oligonucleotides in RNA ID design for characterization of transcripts from linear DNA. This design is used for the study of RNA from premature termination (PT) and RNA from full transcription of the linear template (Figure 1). The oligos are complementary to the region of the circular plasmid (Supplementary Table 1) covering from the promoter to DraIII restriction site.

▲ Complementary DNA oligo ▲ Bit '1' oligo ▲ Repeats label

Table 2

Oligo number	Sequence (5'→ 3')
1	AGCGTGATGCTACTAATTGGGACAATTTTCCAGATGAAGT
2	ATCATCTAAGAATTTAAATGAAGAAGACTTCAGAGCTTTT
3	GTTAAAAATTATTTGGCAAAAATAATATAATTCGGCTGCA
4	GGGGCGGCCTCGTGATACGCCTATTTTTATAGGTTAATGT
5	CATGATAATAATGGTTTCTTAGACGTCAGGTGGCACTTTT
6	CGGGGAAATGTGCGCGGAACCCCTATTTGTTTATTTTTCT
7	AAATACATTCAAATATGTATCCGCTCATGAGACAATAACC
8	CTGATAAATGCTTCAATAATATTGAAAAAGGAAGAGTATG
9	AGTATTCAACATTTCCGTGTCGCCCTTATTCCCTTTTTTG
10	CGGCATTTTGCCTTCCTGTTTTTGCTCACCCAGAAACGCT
11	GGTGAAAGTAAAAGATGCTGAAGATC
12	AGTTGGGTGCACGAGTGGGTTACATCG
13	AACTGGATCTCAACAGCGGTAAGAT
14	CCTTGAGAGTTTTCGCCCCGAAGAACGTTTTCCAATGATG
15	AGCACTTTTAAAGTTCTGCTATGTGGCGCGGTATTATCCC
16	GTATTGACGCCGGGCAAGAGCAACTCGGTCGCCGCATACA
17	CTATTCTCAGAATGACTTGGTTGAGTACTCACCAGTCACA
18	GAAAAGCATCTTACGGATGGCATGACAGTAAGAGAATTAT
19	GCAGTGCTGCCATAACCATGAGTGATAACACTGCGGCCAA
20	CTTACTTCTGACAACGATCGGAGGACCGAAGGAGCTAACC
21	GCTTTTTTGCACAACATGGGGGATCATGTAACTCGCCTTG
22	ATCGTTGGGAACCGGAGCTGAATGAAGCCATACCAAACGA
23	CGAGCGTGACACCACGATGCCTGTAGCAATGGCAACAACG
24	TTGCGCAAACTATTAACTGGCGAACTACTTACTCTAGCTT
25	CCCGGCAACAATTAATAGACTGGATGGAGGCGGATAAAGT
26	TGCAGGACCACTTCTGCGCTCGGCCCTTCCGGCTGGCTGG
27	TTTATTGCTGATAAATCTGGAGCCGGT
28	GAGCGTGGGTCTCGCGGTATCATTGCAG

29	CACTGGGGCCAGATGGTAAGCCCTC
30	CCGTATCGTAGTTATCTACACGACGGGGAGTCAGGCAACT
31	ATGGATGAACGAAATAGACAGATCGCTGAGATAGGTGCCT
32	CACTGATTAAGCATTGGTAACTGTCAGACCAAGTTTACTC
33	ATATATACTTTAGATTGATTTAAAACTTCATTTTTAATTT
34	AAAAGGATCTAGGTGAAGATCCTTTTTGATAATCTCATGA
35	CCAAAATCCCTTAACGTGAGTTTTCGTTCCACTGAGCGTC
36	AGACCCCGTAGAAAAGATCAAAGGATCTTCTTGAGATCCT
37	TTTTTTCTGCGCGTAATCTGCTGCTTGCAAACAAAAAAAC
38	CACCGCTACCAGCGGTGGTTTGTTTGCCGGATCAAGAGCT
39	ACCAACTCTTTTTCCGAAGGTAACTGGCTTCAGCAGAGCG
40	CAGATACCAAATACTGTCCTTCTAGTGTAGCCGTAGTTAG
41	GCCACCACTTCAAGAACTCTGTAGCACCGCCTACATACCT
42	CGCTCTGCTAATCCTGTTACCAGTGGCTGCTGCCAGTGGC
43	GATAAGTCGTGTCTTACCGGGTTGGAC
44	TCAAGACGATAGTTACCGGATAAGGCGC
**45**	AGCGGTCGGGCTGAACGGGGGGTTCTTTGGATATCACTCATTAGTGGT
46	GTGCACACAGCCCAGCTTGGAGCGAACGACCTACACCGAA
47	CTGAGATACCTACAGCGTGAGCTATGAGAAAGCGCCACGC
48	TTCCCGAAGGGAGAAAGGCGGACAGGTATCCGGTAAGCGG
49	CAGGGTCGGAACAGGAGAGCGCACGAGGGAGCTTCCAGGG
50	GGAAACGCCTGGTATCTTTATAGTCCTGTCGGGTTTCGCC
51	ACCTCTGACTTGAGCGTCGATTTTTGTGATGCTCGTCAGG
52	GGGGCGGAGCCTATGGAAAAACGCCAGCAACGCGGCCTTT
53	TTACGGTTCCTGGCCTTTTGCTGGCCTTTTGCTCACATGT
54	TCTTTCCTGCGTTATCCCCTGATTCTGTGGATAACCGTAT
55	TACCGCCTTTGAGTGAGCTGATACCGCTCGCCGCAGCCGA
56	ACGACCGAGCGCAGCGAGTCAGTGAGCGAGGAAGCGGAAG
57	AGCGCCCAATACGCAAACCGCCTCTCCCCGCGCGTTGGCC
58	GATTCATTAATGCAGCTGGCACGACAGGTTTCCCGACTGG
59	AAAGCAATTGGCAGTGAGCGCAACGCA
60	ATTAATGTGAGTTAGCTCACTCATTAGG
**61**	CACCCCAGGCTTTACACTTTATGCTTTTGGATATCACTCATTAGTGGT
62	TCCGGCTCGTATAATGTGTGGAATTATGAGCGGATAATAA
63	TTTCACACAGGAGGTTTAAACTTTAAACATGTCAAAAGAG
64	ACGTCTTTTGTTAAGAATGCTGAGGAACTTGCAAAGCAAA
65	AAATGGATGCTATTAACCCTGAACTTTCTTCAAAATTTAA
66	ATTTTTAATAAAATTCCTGTCTCAGTTTCCTGAAGCTTGC
67	TCTAAACCTCGTTCAAAAAAAAATGCAGAATAAAGTTGGTC
68	AAGAGGAACATATTGAATATTTAGCTCGTAGTTTTCATGA
69	GAGTCGATTGCCAAGAAAACCCACGCCACCTACAACGGTT
70	CCTGATGAGGTGGTTAGCATAGTTCTTAATATAAGTTTTA
71	ATATACAGCCTGAAAATCTTGAGAGAATAAAAGAAGAACA
72	TCGATTTTCCATGGCAGCTGAGAATATTGTAGGAGATCTT
73	CTAGAAAGATTTAAGCCGAGAATGGTCTGTGATCCCCC
74	CATTCCCGGCTACACTGCACCATGATCTTGCTGAAAAACT

**75**	CGAGCCATCCGGAAGATCTGGCGGCCGCTCTCCC
**76**	CAGCAGCAGCAGCAGCAGCAGCAGCAGCAGTTTGGATATCACTCATTAGTGGT/3'-biotin/

Supplementary Table 3.

Sequence of oligonucleotides in RNA ID design for characterization of transcripts from circular DNA. This design is used for the study of RNA IDs from multiple transcription cycles (Figure 3). The oligos are complementary to the entire circular plasmid (Supplementary Table 1). This table includes the same oligonucleotides as Table 2, however it contains 5 extra oligonucelotides (76, 77, 78, 79, 80) which are complementary to the region extending from DraIII restriction site to the T7RNAP promoter.

▲ Complementary DNA oligo ▲ Bit '1' oligo ▲ Repeats label

Table 3

Oligo number	Sequence (5′→ 3′)
1	AGCGTGATGCTACTAATTGGGACAATTTTCCAGATGAAGT
2	ATCATCTAAGAATTTAAATGAAGAAGACTTCAGAGCTTTT
3	GTTAAAAATTATTTGGCAAAAATAATATAATTCGGCTGCA
4	GGGGCGGCCTCGTGATACGCCTATTTTTATAGGTTAATGT
5	CATGATAATAATGGTTTCTTAGACGTCAGGTGGCACTTTT
6	CGGGGAAATGTGCGCGGAACCCCTATTTGTTTATTTTTCT
7	AAATACATTCAAATATGTATCCGCTCATGAGACAATAACC
8	CTGATAAATGCTTCAATAATATTGAAAAAGGAAGAGTATG
9	AGTATTCAACATTTCCGTGTCGCCCTTATTCCCTTTTTTG
10	CGGCATTTTGCCTTCCTGTTTTTGCTCACCCAGAAACGCT
11	GGTGAAAGTAAAAGATGCTGAAGATC
12	AGTTGGGTGCACGAGTGGGTTACATCG
13	AACTGGATCTCAACAGCGGTAAGAT
14	CCTTGAGAGTTTTCGCCCCGAAGAACGTTTTCCAATGATG
15	AGCACTTTTAAAGTTCTGCTATGTGGCGCGGTATTATCCC
16	GTATTGACGCCGGGCAAGAGCAACTCGGTCGCCGCATACA
17	CTATTCTCAGAATGACTTGGTTGAGTACTCACCAGTCACA
18	GAAAAGCATCTTACGGATGGCATGACAGTAAGAGAATTAT
19	GCAGTGCTGCCATAACCATGAGTGATAACACTGCGGCCAA
20	CTTACTTCTGACAACGATCGGAGGACCGAAGGAGCTAACC
21	GCTTTTTTGCACAACATGGGGGATCATGTAACTCGCCTTG
22	ATCGTTGGGAACCGGAGCTGAATGAAGCCATACCAAACGA
23	CGAGCGTGACACCACGATGCCTGTAGCAATGGCAACAACG
24	TTGCGCAAACTATTAACTGGCGAACTACTTACTCTAGCTT
25	CCCGGCAACAATTAATAGACTGGATGGAGGCGGATAAAGT
26	TGCAGGACCACTTCTGCGCTCGGCCCTTCCGGCTGGCTGG
27	TTTATTGCTGATAAATCTGGAGCCGGT

**28**	GAGCGTGGGTCTCGCGGTATCATTGCAG
**29**	CACTGGGGCCAGATGGTAAGCCCTC
**30**	CCGTATCGTAGTTATCTACACGACGGGGAGTCAGGCAACT
**31**	ATGGATGAACGAAATAGACAGATCGCTGAGATAGGTGCCT
**32**	CACTGATTAAGCATTGGTAACTGTCAGACCAAGTTTACTC
**33**	ATATATACTTTAGATTGATTTAAAACTTCATTTTTAATTT
**34**	AAAAGGATCTAGGTGAAGATCCTTTTTGATAATCTCATGA
**35**	CCAAAATCCCTTAACGTGAGTTTTCGTTCCACTGAGCGTC
**36**	AGACCCCGTAGAAAAGATCAAAGGATCTTCTTGAGATCCT
**37**	TTTTTTCTGCGCGTAATCTGCTGCTTGCAAACAAAAAAAC
**38**	CACCGCTACCAGCGGTGGTTTGTTTGCCGGATCAAGAGCT
**39**	ACCAACTCTTTTTCCGAAGGTAACTGGCTTCAGCAGAGCG
**40**	CAGATACCAAATACTGTCCTTCTAGTGTAGCCGTAGTTAG
**41**	GCCACCACTTCAAGAACTCTGTAGCACCGCCTACATACCT
**42**	CGCTCTGCTAATCCTGTTACCAGTGGCTGCTGCCAGTGGC
**43**	GATAAGTCGTGTCTTACCGGGTTGGAC
**44**	TCAAGACGATAGTTACCGGATAAGGCGC
**45**	AGCGGTCGGGCTGAACGGGGGGTTCTTTGGATATCACTCATTAGTGGT
**46**	GTGCACACAGCCCAGCTTGGAGCGAACGACCTACACCGAA
**47**	CTGAGATACCTACAGCGTGAGCTATGAGAAAGCGCCACGC
**48**	TTCCCGAAGGGAGAAAGGCGGACAGGTATCCGGTAAGCGG
**49**	CAGGGTCGGAACAGGAGAGCGCACGAGGGAGCTTCCAGGG
**50**	GGAAACGCCTGGTATCTTTATAGTCCTGTCGGGTTTCGCC
**51**	ACCTCTGACTTGAGCGTCGATTTTTGTGATGCTCGTCAGG
**52**	GGGGCGGAGCCTATGGAAAAACGCCAGCAACGCGGCCTTT
**53**	TTACGGTTCCTGGCCTTTTGCTGGCCTTTTGCTCACATGT
**54**	TCTTTCCTGCGTTATCCCCTGATTCTGTGGATAACCGTAT
**55**	TACCGCCTTTGAGTGAGCTGATACCGCTCGCCGCAGCCGA
**56**	ACGACCGAGCGCAGCGAGTCAGTGAGCGAGGAAGCGGAAG
**57**	AGCGCCCAATACGCAAACCGCCTCTCCCCGCGCGTTGGCC
**58**	GATTCATTAATGCAGCTGGCACGACAGGTTTCCCGACTGG
**59**	AAAGCAATTGGCAGTGAGCGCAACGCA
**60**	ATTAATGTGAGTTAGCTCACTCATTAGG
**61**	CACCCCAGGCTTTACACTTTATGCTTTTGGATATCACTCATTAGTGGT
**62**	TCCGGCTCGTATAATGTGTGGAATTATGAGCGGATAATAA
**63**	TTTCACACAGGAGGTTTAAACTTTAAACATGTCAAAAGAG
**64**	ACGTCTTTTGTTAAGAATGCTGAGGAACTTGCAAAGCAAA
**65**	AAATGGATGCTATTAACCCTGAACTTTCTTCAAAATTTAA
**66**	ATTTTTAATAAAATTCCTGTCTCAGTTTCCTGAAGCTTGC
**67**	TCTAAACCTCGTTCAAAAAAAATGCAGAATAAAGTTGGTC
**68**	AAGAGGAACATATTGAATATTTAGCTCGTAGTTTTCATGA
**69**	GAGTCGATTGCCAAGAAAACCCACGCCACCTACAACGGTT
**70**	CCTGATGAGGTGGTTAGCATAGTTCTTAATATAAGTTTTA
**71**	ATATACAGCCTGAAAATCTTGAGAGAATAAAAGAAGAACA
**72**	TCGATTTTCCATGGCAGCTGAGAATATTGTAGGAGATCTT
**73**	CTAGAAAGATTTAAGCCGAGAATGGTCTGTGATCCCCC

**74**	CATTCCCGGCTACACTGCACCATGATCTTGCTGAAAAACT
**75**	CGAGCCATCCGGAAGATCTGGCGGCCGCTCTCCC
**76**	TATAGTGAGTCGTATTACGCCGGATGGATATGGTGTTC
**77**	AGGCACAAGTGTTAAAGCAGTTGATTTTATTCACTATG
**78**	ATGAAAAAAACAATGAATGGAACCTGCTCCAAGTTA
**79**	AAAATAGAGATAATACCGAAAACTCATCGAGTAGTA
**80**	AGATTAGAGATAATACAACAATAAAAAAATGGTTTAGAACTTACTCACAGC
**81**	CAGCAGCAGCAGCAGCAGCAGCAGCAGCAGTTTGGATATCACTCATTAGTGGT/3’-biotin/

Supplementary Table 4.

Sequence of modified pJET1.2/blunt cloning vector (CloneJET PCR Cloning Kit, Thermo Fisher, Catalog number: K1231): Circular DNA (4527 bp) with inserted $(CTG)_{49}$ tandem repeats. The sequence of the plasmid was verified by whole plasmid sequencing. Map of the plasmid is included at the end of the table.

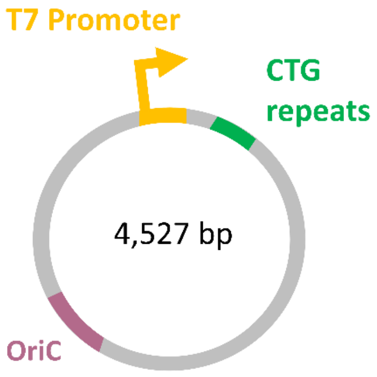

Table 4

Sequence (5′→ 3′)
T7 promoter $(CTG)_{49}$ repeats   OriC   DraIII Cutting
GCATCCATTTTTTGCTTTGCAAGTTCCTCAGCATTCTTAACAAAAGACGTCTCTTTTGACATGTTT
AAAGTTTAAACCTCCTGTGTGAAATTATTATCCGCTCATAATTCCACACATTATACTGAGAGATCC
CCTCATAATTTCCCCAAAGCGTAACCATGTGTGAATAAATTTTGAGCTAGTAGGGTTGCAGCCACG
AGTAAGTCTTCCCTTGTTATTGTGTAGCCAGAATGCCGCAAAACTTCCATGCCTAAGCGAACTGTT
GAGAGTACGTTTCGATTTCTGACTGTGTTAGCCTGGAAGTGCTTGTCCCAACCTTGTTTCTGAGCA
TGAACGCCCGCAAGCCAACATGTTAGTTGAAGCATCAGGGCGATTAGCAGCATGATATCAAAACGC
TCTGAGCTGCTCGTTCGGCTATGGCGTAGGCCTAGTCCGTAGGCAGGACTTTTCAAGTCTCGGAAG
GTTTCTTCAATCTGCATTCGCTTCGAATAGATATTAACAAGTTGTTTGGGTGTTCGAATTTCAACA
GGTAAGTTAGTTGCTAGAACCCATGGCTCCTTTGCCGACGCTGAGTAGATTTTAGGTGACGGGTGG
TGACAATGAGTCCGTGTCGAGCGCTGATTTTTTCGGCCTTTAGAGCGAGATTTATACAATAGAATT
TGGCATGAGATTGGATTGCTTTTAGTCAGCCTCTTATAGCCTAAAGTCTTTGAGTGACTAGATGAC
ATATCATGTAAGTTGCTGATAGGTTTCCAGTTTTTCCGCTCCTAGGTCTGCATATTGTACTTTTCC
TCTTACTCGACTTAACCAGTACCAACCCAGCTTCTCAACGGATTTATACCATGGCACTTTAAAGCC
AGCATCACTGACAATGAGCGGTGTGGTGTTACTCGGTAGAATGCTCGCAAGGTCGGCTAGAAATTG
GTCATGAGCTTTCTTTGAACATTGCTCTGAAAGCGGGAACGCTTTCTCATAAAGAGTAACAGAACG
ACCGTGTAGTGCGACTGAAGCTCGCAATACCATAAGTCGTTTTTGCTCACGAATATCAGACCAGTC
AACAAGTACAATGGGCATCGTATTGCCCGAACAGATAAAGCTAGCATGCCAACGGTATACAGCGAG
TCGCTCTTTGTGGAGGTGACGATTACCTAACAATCGGTCGATTCGTTTGATGTTATGTTTTGTTCT
CGCTTTGGTTGGCAGGTTACGGCCAAGTTCGGTAAGAGTGAGAGTTTTACAGTCAAGTAATGCGTG
GCAAGCCAACGTTAAGCTGTTGAGTCGTTTTAAGTGTAATTCGGGGCAGAATTGGTAAAGAGAGTC
GTGTAAAATATCGAGTTCGCACATCTTGTTGTCTGATTATTGATTTTTCGCGAAACCATTTGATCA

TATGACAAGATGTGTATCCACCTTAACTTAATGATTTTTACCAAAATCATTAGGGGATTCATCAGC
ACATTATACGAGCCGGAAGCATAAAGTGTAAAGCCTGGGGTGCCTAATGAGTGAGCTAACTCACAT
TAATTGCGTTGCGCTCACTGCCAATTGCTTTCCAGTCGGGAAACCTGTCGTGCCAGCTGCATTAAT
GAATCGGCCAACGCGCGGGGAGAGGCGGTTTGCGTATTGGGCGCTCTTCCGCTTCCTCGCTCACTG
ACTCGCTGCGCTCGGTCGTTCGGCTGCGGCGAGCGGTATCAGCTCACTCAAAGGCGGTAATACGGT
TATCCACAGAATCAGGGGATAACGCAGGAAAGAACATGTGAGCAAAAGGCCAGCAAAAGGCCAGGA
ACCGTAAAAAGGCCGCGTTGCTGGCGTT**TTTCCATAGGCTCCGCCCCCCTGACGAGCATCACAAAA**
ATCGACGCTCAAGTCAGAGGTGGCGAAACCCGACAGGACTATAAAGATACCAGGCGTTTCCCCCTG
GAAGCTCCCTCGTGCGCTCTCCTGTTCCGACCCTGCCGCTTACCGGATACCTGTCCGCCTTTCTCC
CTTCGGGAAGCGTGGCGCTTTCTCATAGCTCACGCTGTAGGTATCTCAGTTCGGTGTAGGTCGTTC
GCTCCAAGCTGGGCTGTGTGCACGAACCCCCCGTTCAGCCCGACCGCTGCGCCTTATCCGGTAACT
ATCGTCTTGAGTCCAACCCGGTAAGACACGACTTATCGCCACTGGCAGCAGCCACTGGTAACAGGA
TTAGCAGAGCGAGGTATGTAGGCGGTGCTACAGAGTTCTTGAAGTGGTGGCCTAACTACGGCTACA
CTAGAAGAACAGTATTTGGTATCTGCGCTCTGCTGAAGCCAGTTACCTTCGGAAAAAGAGTTGGTA
GCTCTTGATCCGGCAAACAAACCACCGCTGGTAGCGGTGGTTTTTTTGTTTGCAAGCAGCAGATTA
CGCGCAGAAAAAAAGGATCTCAAGAAGATCCTTTGATCTTTTCTACGGGGTCTGACGCTCAGTGGA
ACGAAAACTCACGTTAAGGGATTTTGGTCATGAGATTATCAAAAAGGATCTTCACCTAGATCCTTT
TAAATTAAAAATGAAGTTTTAAATCAATCTAAAGTATATATGAGTAAACTTGGTCTGACAGTTACC
AATGCTTAATCAGTGAGGCACCTATCTCAGCGATCTGTCTATTTCGTTCATCCATAGTTGCCTGAC
TCCCCGTCGTGTAGATAACTACGATACGGGAGGGCTTACCATCTGGCCCCAGTGCTGCAATGATAC
CGCGAGACCCACGCTCACCGGCTCCAGATTTATCAGCAATAAACCAGCCAGCCGGAAGGGCCGAGC
GCAGAAGTGGTCCTGCAACTTTATCCGCCTCCATCCAGTCTATTAATTGTTGCCGGGAAGCTAGAG
TAAGTAGTTCGCCAGTTAATAGTTTGCGCAACGTTGTTGCCATTGCTACAGGCATCGTGGTGTCAC
GCTCGTCGTTTGGTATGGCTTCATTCAGCTCCGGTTCCCAACGATCAAGGCGAGTTACATGATCCC
CCATGTTGTGCAAAAAAGCGGTTAGCTCCTTCGGTCCTCCGATCGTTGTCAGAAGTAAGTTGGCCG
CAGTGTTATCACTCATGGTTATGGCAGCACTGCATAATTCTCTACTGTCATGCCATCCGTAAGATG
CTTTTCTGTGACTGGTGAGTACTCAACCAAGTCATTCTGAGAATAGTGTATGCGGCGACCGAGTTG
CTCTTGCCCGGCGTCAATACGGGATATAACCGCGCCACAGTAGCAGAGCTTTAAAAGTGCTCATCA
TTGGAAAACGTTCTTCGGGGCGGAAAACTCTTCAAGGATCTTACCGCTGTTGAGATCCAGTTCGAT
GTAACCCACTCGTGAACCGAACTGATCTTCGTGCATATTTTACCGCCTTTCCCAGCGTTTCTGGGT
CAAACCTTCTTAAAAACAGGAAGGCAAAATGCCGCAAAAAAGGGAATAAGGGCGACACGGAAATGT
TGAATACTCATACTCTTCCTTTTTCAATATTATTGAAGCATTTATCAGGGTTATTGTCTCATGAGC
GGATACATATTTGAATGTATTTAGAAAAATAAACAAATAGGGGTTCCGCGCACATTTCCCCGAAAA
GTGCCACCTGACGTCTAAGAAACCATTATTATCATGACATTAACCTATAAAAATAGGCGTATCACG
AGGCCGCCCCTGCAGCCGAATTATATTATTTTTGCCAAATAATTTTTAACAAAAGCTCTGAAGTCT
TCTTCATTTAAATTCTTAGATGATACTTCATCTGGAAAATTGTCCCAATTAGTAGCAT**CACGCTGT**
GAGTAAGTTCTAAACCATTTTTTTTATTGTTGTATTATCTCTAATCTTACTACTCGATGAGTTTTC
GGTATTATCTCTATTTTTAACTTGGAGCAGGTTCCATTCATTGTTTTTTTCATCATAGTGAATAAA
ATCAACTGCTTTAACACTTGTGCCTGAACACCATATCCATCCGGCG**TAATACGACTCACTATAGG**G
AGAGCGGCCGCCAGATCTTCCGGATGGCTCGAGTTTTTCAGCAAGATCATGGTGCAGTGTAGCCGG
GAATG**CTGCTGCTGCTGCTGCTGCTGCTGCTGCTGCTGCTGCTGCTGCTGCTGCTGCTGCTGCTGC**
TGCTGCTGCTGCTGCTGCTGCTGCTGCTGCTGCTGCTGCTGCTGCTGCTGCTGCTGCTGCTGCTGC
TGCTGCTGCTGCTGCTGCTGGGGGGGATCACAGACCATTCTCGGCTTAAATCTTTCTAGAAGATCT
CCTACAATATTCTCAGCTGCCATGGAAAATCGATGTTCTTCTTTTATTCTCTCAAGATTTTCAGGC
TGTATATTAAAACTTATATTAAGAACTATGCTAACCACCTCATCAGGAACCGTTGTAGGTGGCGTG
GGTTTTCTTGGCAATCGACTCTCATGAAAACTACGAGCTAAATATTCAATATGTTCCTCTTGACCA
ACTTTATTCTGCATTTTTTTTGAACGAGGTTTAGAGCAAGCTTCAGGAAACTGAGACAGGAATTTT
ATTAAAAATTTAAATTTTGAAGAAAGTTCAGGGTTAATA

Map of the plasmid showing restriction sites, T7RNAP promoter sequence, the OriC and the CTG repeats.

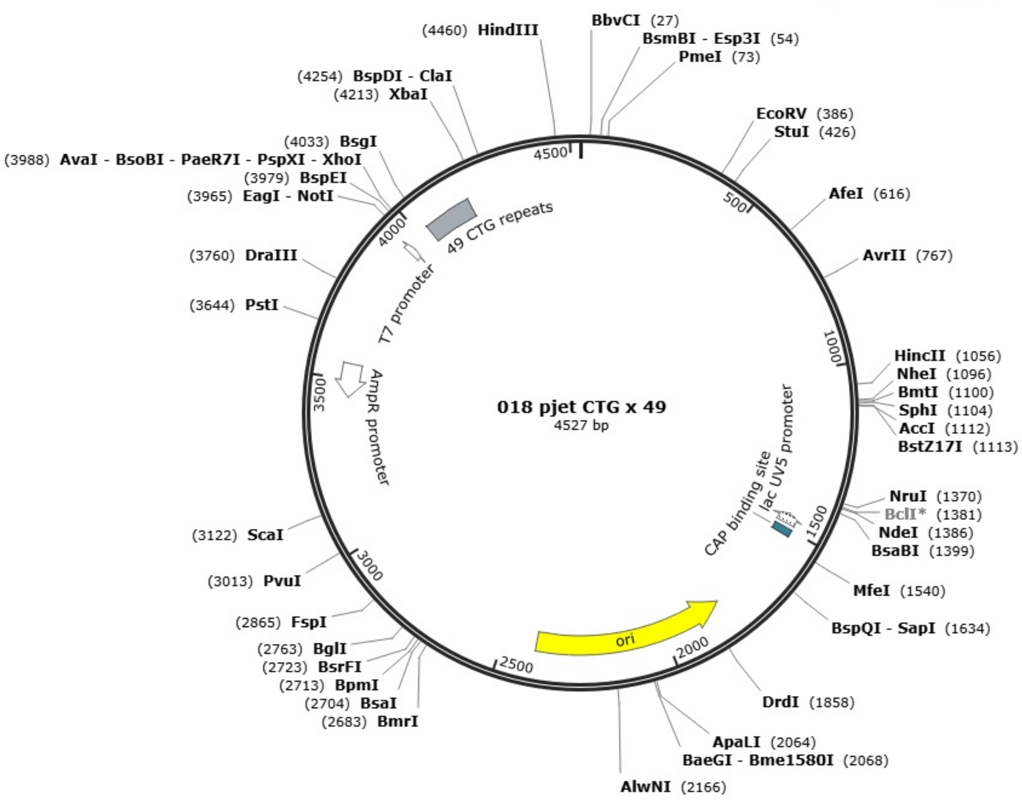

Supplementary Table 5.

PFGG plasmid. The plasmid was produced by IDT and verified using next-generation sequencing and Sanger sequencing.

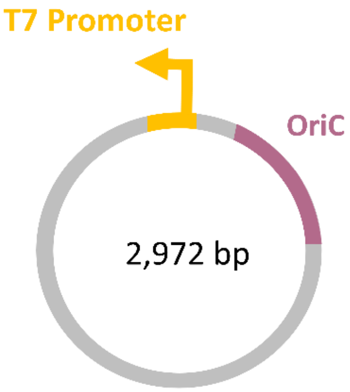

Table 5

Sequence (5′→ 3′)
T7 promoter OriC ScaI cutting
TCGCGCGTTTCGGTGATGACGGTGAAAACCTCTGACACATGCAGCTCCCCTAGACGGTCACAGCTT
GTCTGTAAGCGGATGCCGGGAGCAGACAAGCCCGTCAGGGCGCGTCAGCGGGTGTTGGCGGGTGTC
GGGGCTGGCTTAACTATGCGGCATCAGAGCAGATTGTACTGAGAGTGCACCAAATGCGGTGTGAAA
TACCGCACAGATGCGTAAGGAGAAAATACCGCATCAGGCGCCATTCGCCATTCAGGCTGCGCAACT
GTTGGGAAGGGCGATCGGTGCGGGCCTCATCGCTATTACGCCAGCTGGCGAAAGGGGGATGTGCTG
CAAGGCGATTAAGTTGGGTAACGCCAGGGTTTTCCCAGTCACGACGTTGTAAAACGACGGCCAGTG
CAACGCGATGACGATGGATAGCGATTCATCGATGAGCTGACCCGATCGCCGCCGCCGGAGGGTTGC
GTTTGAGACAGGCGACAGATCCTGCAGGAAGGTTTAAACGCATTTAGGTGACACTATAGAAGTGGA
ATCCGCTCGAGGGATCCGAATTCGAAGCTTTGGTACAATTCGAGACCGGAGCGAGACGGGAGTCCA
GATCTCCATCGTCTCACCATGGTCTCAAGCTACCTGAAGCTTTCTTAATTAAGACGTCAGAATTCT
CGAGGCGGCCGCATGTGAGTCTC**CCTATAGTGAGTCGTATTA**ATCAGTTCTGGACCAGCGAGCTGT
GCTGCGACTCGTGGCGTAATCATGGTCATAGCTGTTTCCTGTGTGAAATTGTTATCCGCTCACAAT
TCCACACAACATACGAGCCGGAAGCATAAAGTGTAAAGCCTGGGGTGCCTAATGAGTGAGCTAACT
CACATTAATTGCGTTGCGCTCACTGCCCGCTTTCCAGTCGGGAAACCTGTCGTGCCAGCTGCATTA
ATGAATCGGCCAACGCGCGGGGAGAGGCGGTTTGCGTATTGGGCGCTCTTCCGCTTCCTCGCTCAC
TGACTCGCTGCGCTCGGTCGTTCGGCTGCGGCGAGCGGTATCAGCTCACTCAAAGGCGGTAATACG
GTTATCCACAGAATCAGGGGATAACGCAGGAAAGAACATGTGAGCAAAAGGCCAGCAAAAGGCCAG
GAACCGTAAAAAGGCCGCGTTGCTGGCGTT**TTTCCATAGGCTCCGCCCCCCTGACGAGCATCACAA**
**AAATCGACGCTCAAGTCAGAGGTGGCGAAACCCGACAGGACTATAAAGATACCAGGCGTTTCCCCC**
**TGGAAGCTCCCTCGTGCGCTCTCCTGTTCCGACCCTGTCGCTTACCGGATACCTGTCCGCCTTTCT**
**CCCTTCGGGAAGCGTGGCGCTTTCTCATAGCTCACGCTGTAGGTATCTCAGTTCGGTGTAGGTCGT**
**TCGCTCCAAGCTGGGCTGTGTGCACGAACCCCCCGTTCAGCCCGACCGCTGCGCCTTATCCGGTAA**
**CTATCGTCTTGAGTCCAACCCGGTAAGACACGACTTATCGCCACTGGCAGCAGCCACTGGTAACAG**
**GATTAGCAGAGCGAGGTATGTAGGCGGTGCTACAGAGTTCTTGAAGTGGTGGCCTAACTACGGCTA**

```
CACTAGAAGAACAGTATTTGGTATCTGCGCTCTGCTGAAGCCAGTTACCTTCGGAAAAAGAGTTGG
TAGCTCTTGATCCGGCAAACAAACCACCGCTGGTAGCGGTGGTTTTTTTGTTTGCAAGCAGCAGAT
TACGCGCAGAAAAAAAGGATCTCAAGAAGATCCTTTGATCTTTTCTACGGGGTCTGACGCTCAGTG
GAACGAAAACTCACGTTAAGGGATTTTGGTCATGAGATTATCAAAAAGGATCTTCACCTAGATCCT
TTTAAATTAAAAATGAAGTTTTAAATCAATCTAAAGTATATATGAGTAAACTTGGTCTGACAGTTA
CCAATGCTTAATCAGTGAGGCACCTATCTCAGCGATCTGTCTATTTCGTTCATCCATAGTTGCCTG
ACTCCCCGTCGTGTAGATAACTACGATACGGGAGGGCTTACCATCTGGCCCCAGTGCTGCAATGAT
ACCGCGAGATCCACGCTCACCGGCTCCAGATTTATCAGCAATAAACCAGCCAGCCGGAAGGGCCGA
GCGCAGAAGTGGTCCTGCAACTTTATCCGCCTCCATCCAGTCTATTAATTGTTGCCGGGAAGCTAG
AGTAAGTAGTTCGCCAGTTAATAGTTTGCGCAACGTTGTTGCCATTGCTACAGGCATCGTGGTGTC
ACGCTCGTCGTTTGGTATGGCTTCATTCAGCTCCGGTTCCCAACGATCAAGGCGAGTTACATGATC
CCCCATGTTGTGCAAAAAAGCGGTTAGCTCCTTCGGTCCTCCGATCGTTGTCAGAAGTAAGTTGGC
CGCAGTGTTATCACTCATGGTTATGGCAGCACTGCATAATTCTCTTACTGTCATGCCATCCGTAAG
ATGCTTTTCTGTGACTGGTGAGTACTCAACCAAGTCATTCTGAGAATAGTGTATGCGGCGACCGAG
TTGCTCTTGCCCGGCGTCAATACGGGATAATACCGCGCCACATAGCAGAACTTTAAAAGTGCTCAT
CATTGGAAAACGTTCTTCGGGGCGAAAACTCTCAAGGATCTTACCGCTGTTGAGATCCAGTTCGAT
GTAACCCACTCGTGCACCCAACTGATCTTCAGCATCTTTTACTTTCACCAGCGTTTCTGGGTGAGC
AAAAACAGGAAGGCAAAATGCCGCAAAAAAGGGAATAAGGGCGACACGGAAATGTTGAATACTCAT
ACTCTACCTTTTTCAATATTATTGAAGCATTTATCAGGGTTATTGTCTCATGAGCGGATACATATT
TGAATGTATTTAGAAAAATAAACAAATAGGGGTTCCGCGCACATTTCCCCGAAAAGTGCCACCTGA
CGTCTAAGAAACCATTATTATCATGACATTAACCTATAAAAATAGGCGTATCACGAGGCCCTTTCA
TC
```

Map of the plasmid showing restriction sites, T7RNAP promoter sequence and the OriC.

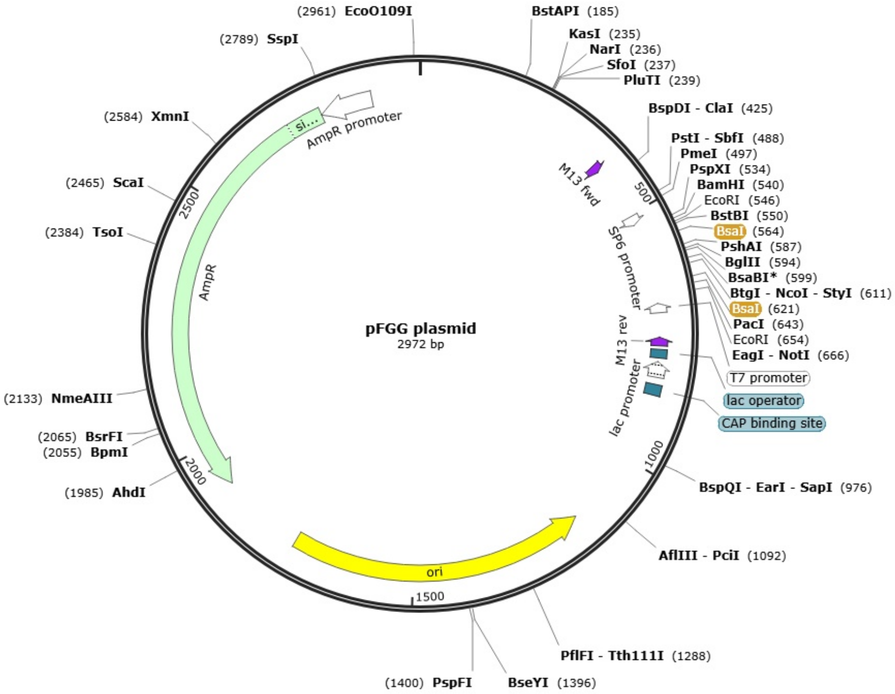

Supplementary Table 6.

Sequence of oligonucleotides used to assemble RNA ID for characterization of transcripts originating from 4.5 kbp DNA construct. This construct has a larger separation between T7 promoter and OriC (Supplementary Table 4). This design is used for the experiments presented in Supplementary Figure 25.

Table 6

Oligo number	Sequence (5′→ 3′)
1	AGCGTGATGCTACTAATTGGGACAATTTTCCAGATGAAGT
2	ATCATCTAAGAATTTAAATGAAGAAGACTTCAGAGCTTTT
3	GTTAAAAATTATTTGGCAAAAATAATATAATTCGGCTGCA
4	GGGGCGGCCTCGTGATACGCCTATTTTTATAGGTTAATGT
5	CATGATAATAATGGTTTCTTAGACGTCAGGTGGCACTTTT
6	CGGGGAAATGTGCGCGGAACCCCTATTTGTTTATTTTTCT
7	AAATACATTCAAATATGTATCCGCTCATGAGACAATAACC
8	CTGATAAATGCTTCAATAATATTGAAAAAGGAAGAGTATG
9	AGTATTCAACATTTCCGTGTCGCCCTTATTCCCTTTTTTG
10	CGGCATTTTGCCTTCCTGTTTTTAAGAAGGTTTGACCCAG
11	AAACGCTGGGAAAGGCGGTAAAATATGCACGAAGATCAGT
12	TCGGTTCACGAGTGGGTTACATCGAACTGGATCTCAACAG
13	CGGTAAGATCCTTGAAGAGTTTTCCGCCCCGAAGAACGTT
14	TTCCAATGATGAGCACTTTTAAAGCTCTGCTACTGTGGCG
15	CGGTTATATCCCGTATTGACGCCGGGCAAGAGCAACTCGG
16	TCGCCGCATACACTATTCTCAGAATGACTTGGTTGAGTAC
17	TCACCAGTCACAGAAAAGCATCTTACGGATGGCATGACAG
18	TAGAGAATTATGCAGTGCTGCCATAACCATGAGTGATAAC
19	ACTGCGGCCAACTTACTTCTGACAACGATCGGAGGACCGA
20	AGGAGCTAACCGCTTTTTTGCACAACATGGGGGATCATGT
21	AACTCGCCTTGATCGTTGGGAACCGGAGCTGAATGAAGCC
22	ATACCAAACGACGAGCGTGACACCACGATGCCTGTAGCAA
23	TGGCAACAACGTTGCGCAAACTATTAACTGGCGAACTACT
24	TACTCTAGCTTCCCGGCAACAATTAATAGACTGGATGGAG
25	GCGGATAAAGTTGCAGGACCACTTCTGCGCTCGGCCCTTC
26	CGGCTGGCTGGTTTATTGCTGATAAATCTGGAGCCGGTGA
27	GCGTGGGTCTCGCGGTATCATTGCAGCACTGGGGCCAGAT
28	GGTAAGCCCTCCCGTATCGTAGTTATCTACACGACGGGGA
29	GTCAGGCAACTATGGATGAACGAAATAGACAGATCGCTGA
30	GATAGGTGCCTCACTGATTAAGCATTGGTAACTGTCAGAC
31	CAAGTTTACTCATATATACTTTAGATTGATTTAAAACTTC
32	ATTTTTAATTTAAAAGGATCTAGGTGAAGATCCTTTTTGA
33	TAATCTCATGACCAAAATCCCTTAACGTGAGTTTTCGTTC
34	CACTGAGCGTCAGACCCCGTAGAAAAGATCAAAGGATCTT
35	CTTGAGATCCTTTTTTTCTGCGCGTAATCTGCTGCTTGCA
36	AACAAAAAAACCACCGCTACCAGCGGTGGTTTGTTTGCCG

37	GATCAAGAGCTACCAACTCTTTTTCCGAAGGTAACTGGCT
38	TCAGCAGAGCGCAGATACCAAATACTGTTCTTCTAGTGTA
39	GCCGTAGTTAGGCCACCACTTCAAGAACTCTGTAGCACCG
40	CCTACATACCTCGCTCTGCTAATCCTGTTACCAGTGGCTG
41	CTGCCAGTGGCGATAAGTCGTGTCTTACCGGGTTGGACTC
42	AAGACGATAGTTACCGGATAAGGCGCAGCGGTCGGGCTGA
43	ACGGGGGGTTCGTGCACACAGCCCAGCTTGGAGCGAACGA
44	CCTACACCGAACTGAGATACCTACAGCGTGAGCTATGAGA
45	AAGCGCCACGCTTCCCGAAGGGAGAAAGGCGGACAGGTAT
46	CCGGTAAGCGGCAGGGTCGGAACAGGAGAGCGCACGAGGG
47	AGCTTCCAGGGGGAAACGCCTGGTATCTTTATAGTCCTGT
48	CGGGTTTCGCCACCTCTGACTTGAGCGTCGATTTTTGTGA
49	TGCTCGTCAGGGGGGCGGAGCCTATGGAAAAACGCCAGCA
50	ACGCGGCCTTTTTACGGTTCCTGGCCTT
51	TTGCTGGCCTTTTGCTCACATGTTCTTTC
52	CTGCGTTATCCCCTGATTCTGTGGATTTGGATATCACTCATTAGTGGT
53	TAACCGTATTACCGCCTTTGAGTGAGCTGATACCGCTCGC
54	CGCAGCCGAACGACCGAGCGCAGCGAGTCAGTGAGCGAGG
55	AAGCGGAAGAGCGCCCAATACGCAAACCGCCTCTCCCCGC
56	GCGTTGGCCGATTCATTAATGCAGCTGGCACGACAGGTTT
57	CCCGACTGGAAAGCAATTGGCAGTGAGCGCAACGCAATTA
58	ATGTGAGTTAGCTCACTCATTAGGCACCCCAGGCTTTACA
59	CTTTATGCTTCCGGCTCGTATAATGTGCTGATGAATCCCC
60	TAATGATTTTGGTAAAAATCATTAAGTTAAGGTGGATACA
61	CATCTTGTCATATGATCAAATGGTTTCGCGAAAAATCAAT
62	AATCAGACAACAAGATGTGCGAACTCGATATTTTACACGA
63	CTCTCTTTACCAATTCTGCCCCGAATTACACTTAAAACGA
64	CTCAACAGCTTAACGTTGGCTTGCCACGCATTACTTGACT
65	GTAAAACTCTCACTCTTACCGAACTTGGCCGTAACCTGCC
66	AACCAAAGCGAGAACAAAACATAACATCAAACGAATCGAC
67	CGATTGTTAGGTAATCGTCACCTCCACAAAGAGCGACTCG
68	CTGTATACCGTTGGCATGCTAGCTTTATCTGTTCGGGCAA
69	TACGATGCCCATTGTACTTGTTGACTGGTCTGATATTCGT
70	GAGCAAAAACGACTTATGGTATTGCGAGCTTCAGTCGCAC
71	TACACGGTCGTTCTGTTACTCTTTATGAGAAAGCGTTCCC
72	GCTTTCAGAGCAATGTTCAAAGAAAGCTCATGACCAATTT
73	CTAGCCGACCTTGCGAGCATTCTACCGAGTAACACCACAC
74	CGCTCATTGTCAGTGATGCTGGCTTTAAAGTGCCA
75	TGGTATAAATCCGTTGAGAAGCTGGTTTGGATATCACTCATTAGTGGT
76	GTTGGTACTGGTTAAGTCGAGTAAGAGGAAAAGTACAATA
77	TGCAGACCTAGGAGCGGAAAAACTGGAAACCTATCAGCAA
78	CTTACATGATATGTCATCTAGTCACTCAAAGACTTTAGGC
79	TATAAGAGGCTGACTAAAAGCAATCCAATCTCATGCCAAA
80	TTCTATTGTATAAATCTCGCTCTAAAGGCCGAAAAAATCA
81	GCGCTCGACACGGACTCATTGTCACCACCCGTCACCTAAA
82	ATCTACTCAGCGTCGGCAAAGGAGCCATGGGTTCTAGCAA

83	CTAACTTACCTGTTGAAATTCGAACACCCAAACAACTTGT
84	TAATATCTATTCGAAGCGAATGCAGATTGAAGAAACCTTC
85	CGAGACTTGAAAAGTCCTGCCTACGGACTAGGCCTACGCC
86	ATAGCCGAACGAGCAGCTCAGAGCGTTTTGATATCATGCT
87	GCTAATCGCCCTGATGCTTCAACTAACATGTTGGCTTGCG
88	GGCGTTCATGCTCAGAAACAAGGTTGGGACAAGCACTTCC
89	AGGCTAACACAGTCAGAAATCGAAACGTACTCTCAACAGT
90	TCGCTTAGGCATGGAAGTTTTGCGGCATTCTGGCTACACA
91	ATAACAAGGGAAGACTTACTCGTGGCTGCAACCCTACTAG
92	CTCAAAATTTATTCACACATGGTTACGCTTTGGGGAAATT
93	ATGAGGGGATCTCTCAGTATAATGTGTGGAATTATGAGCG
94	GATAATAATTTCACACAGGAGGTTTAAACTTTAAACATGT
95	CAAAAGAGACGTCTTTTGTTAAGAATGCTGAGGAACTTGC
96	AAAGCAAAAAAATGGATGCTATTAACCCTGAACTTTCTTCA
97	AAATTTAAATTTTTAATAAAATTCCTGTCTCAGTT
98	TCCTGAAGCTTGCTCTAAACCTCGTTTTGGATATCACTCATTAGTGGT
99	TCAAAAAAAATGCAGAATAAAGTTGTTTGGATATCACTCATTAGTGGT
100	GTCAAGAGGAACATATTGAATATTTAGCTCGTAGTTTTCA
101	TGAGAGTCGATTGCCAAGAAAACCCACGCCACCTACAACG
102	GTTCCTGATGAGGTGGTTAGCATAGTTCTTAATATAAGTT
103	TTAATATACAGCCTGAAAATCTTGAGAGAATAAAAGAAGA
104	ACATCGATTTTCCATGGCAGCTGAGAATATTGTAGGAGAT
105	CTTCTAGAAAGATTTAAGCCG
106	AGAATGGTCTGTGATCCCCCC
107	CATTCCCGGCTACACTGCACCATGATCTTGCTGAAAAACT
108	CGAGCCATCCGGAAGATCTGGCGGCCGCTCTCCC
109	CAGCAGCAGCAGCAGCAGCAGCAGCAGCAG

Supplementary Table 7.

Identified sequence within DNA construct that shows structural similarity to engineered T7RNAP transcription terminators.

Table 7

DNA	Sequence (5′→ 3′)
1	CAAACAAACCACCGCTGGTAGCGGTGGTTTTTTTGTTT

Supplementary Table 8.

The table shows the IV curves of nanopores used to study RNA IDs. The ionic current and RMS noise at 600 mV is presented for each pore. An estimate of each nanopore diameter was calculated from the ionic current values presented, assuming a conical pore geometry[4].

Table 8

Pore number	Sample	Current, RMS noise at 600 mV and calculated pore diameter	Current / voltage curve (IV curve)
1	RNA IDs produced from linear DNA	11.4 nA<br>6.5 pA<br>~8 nm	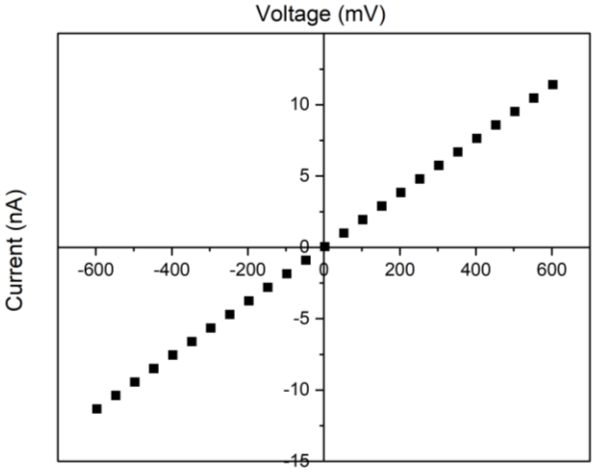
2	RNA IDs produced from linear DNA, and DNA template	9.9 nA<br>6.7 pA<br>~7 nm	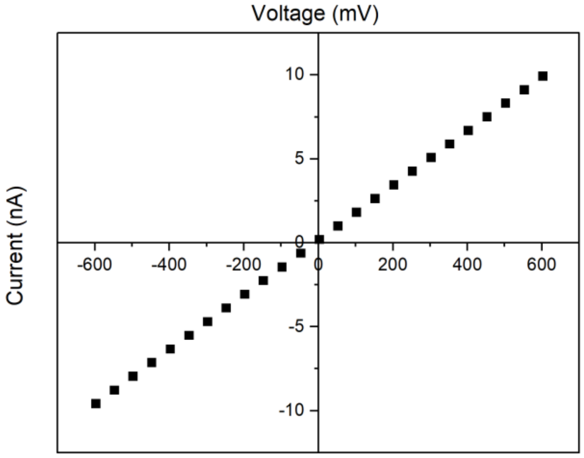

3	RNA IDs<br>of produced<br>from circular<br>template	8.7 nA<br>6.2 pA<br>~6 nm	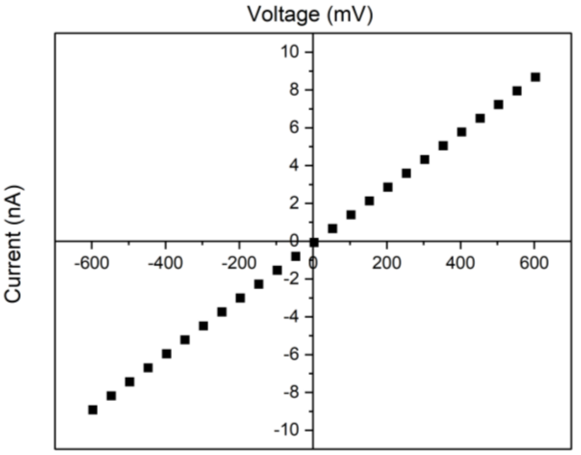
4	RNA IDs<br>of produced<br>from circular<br>template	7.5 nA<br>6.6 pA<br>~5 nm	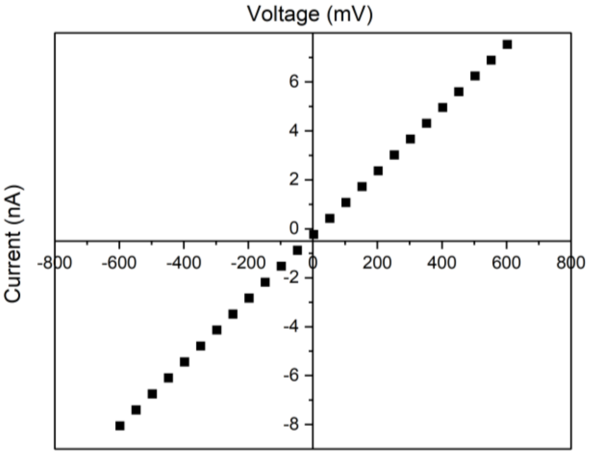
5	RNA IDs<br>of produced<br>from circular<br>template	12.2 nA<br>6.4 pA<br>~9 nm	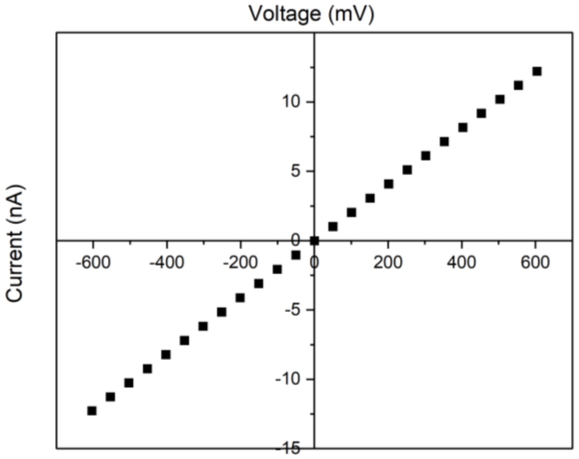

6	RNA IDs of produced from circular template	10.0 nA<br>6.4 pA<br>~7 nm	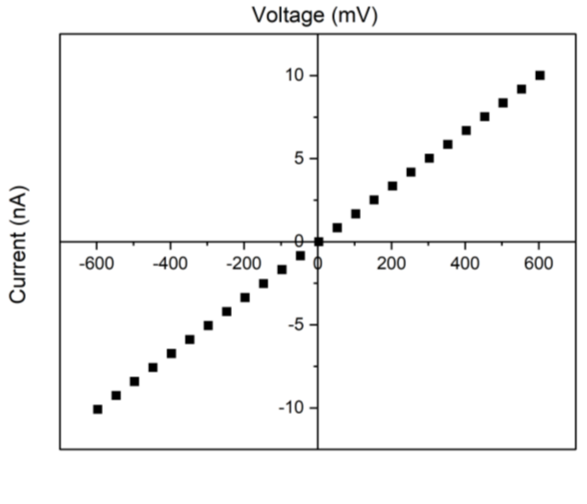
7	DNA ladder SF8 (0.5 kbp – 10 kbp)	11.1 nA<br>5.7 pA<br>~8 nm	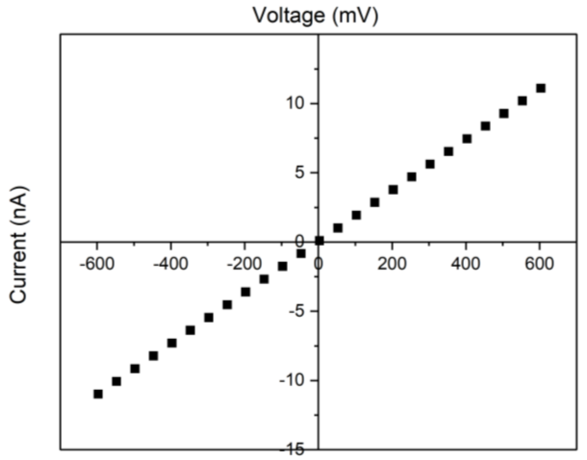
8	RNA IDs produced from 4.5 kbp DNA	10.9 nA<br>6.4 pA<br>~8 nm	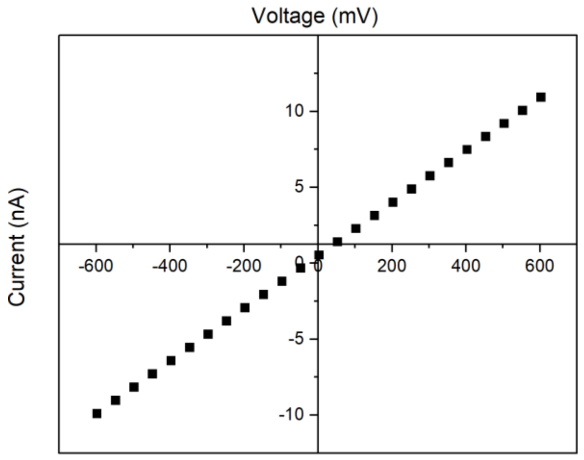

An estimate the pore's diameter was calculated from the overall resistance of the nanopore in open state, R, which is constituted by the resistance of the pore cavity region, R_{pore}, and the resistance of the access region of the pore, R_{acc}[4]:

$$R = R_{pore} + R_{acc}$$

This equation can be rewritten in terms of the resistivity ρ of the electrolytic solution, the pore's length, L, and the diameter of the *cis* and *trans* aperture of the pore, D_{cis} and D_{trans} :

$$R = \rho \frac{4L}{\pi D_{trans} D_{cis}} + \rho \left(\frac{1}{2\, D_{trans}} + \frac{1}{2\, D_{cis}}\right)$$

The diameter of the pore D_{cis} was calculated using the experimental ionic current I during the application of a 600 mV potential, assuming a D_{trans} of 200 µm, conductivity of $15.5\ Sm^{-1}$ for 4M LiCl, and length L of 950 µm for our glass nanopores [5,6].

Supplementary References

1. Chen, K. et al. Dynamics of driven polymer transport through a nanopore. Nat Phys **17**, 1043–1049 (2021).

2. Bell, N. A. W., Muthukumar, M. & Keyser, U. F. Translocation frequency of double-stranded DNA through a solid-state nanopore. *Phys Rev E* **93**, 022401 (2016).

3. Wanunu, M., Morrison, W., Rabin, Y., Grosberg, A. Y. & Meller, A. Electrostatic focusing of unlabelled DNA into nanoscale pores using a salt gradient. *Nat Nanotechnol* **5**, 160–165 (2010).

4. Tang, W., Fried, J. P., Tilley, R. D. & Gooding, J. J. Understanding and modelling the magnitude of the change in current of nanopore sensors. Chem Soc Rev **51**, 5757–5776 (2022).

5. Lin, Y. et al. Characterization of DNA duplex unzipping through a sub-2 nm solid-state nanopore. Chemical Communications **53**, 3539–3542 (2017).

6. Bell, N. A. W. & Keyser, U. F. Digitally encoded DNA nanostructures for multiplexed, single-molecule protein sensing with nanopores. Nat Nanotechnol **11**, 645–651 (2016).
